# Novel gold(III)-dithiocarbamate complex targeting bacterial thioredoxin reductase: antimicrobial activity, synergy, toxicity, and mechanistic insights

**DOI:** 10.3389/fmicb.2023.1198473

**Published:** 2023-06-02

**Authors:** Carlos Ratia, Victoria Ballén, Yaiza Gabasa, Raquel G. Soengas, María Velasco-de Andrés, María José Iglesias, Qing Cheng, Francisco Lozano, Elias S. J. Arnér, Fernando López-Ortiz, Sara M. Soto

**Affiliations:** ^1^Barcelona Institute for Global Health (ISGlobal), Universitat de Barcelona, Barcelona, Spain; ^2^Área de Química Orgánica, Centro de Investigación CIAIMBITAL, Universidad de Almería, Almería, Spain; ^3^August Pi i Sunyer Biomedical Research Institute (IDIBAPS), Barcelona, Spain; ^4^Division of Biochemistry, Department of Medical Biochemistry and Biophysics, Karolinska Institutet, Stockholm, Sweden; ^5^Servei d’Immunologia, Centre de Diagnòstic Biomèdic, Hospital Clínic de Barcelona, Barcelona, Spain; ^6^Department de Biomedicina, Facultat de Medicina, Universitat de Barcelona, Barcelona, Spain; ^7^Department of Selenoprotein Research and the National Tumor Biology Laboratory, Budapest, Hungary; ^8^CIBER Enfermedades Infecciosas (CIBERINFEC), Instituto de Salud Carlos III, Madrid, Spain

**Keywords:** cycloaurate, dithiocarbamate, MDR, MRSA, synergy, gold(III) complex

## Abstract

**Introduction:**

Antimicrobial resistance is a pressing global concern that has led to the search for new antibacterial agents with novel targets or non-traditional approaches. Recently, organogold compounds have emerged as a promising class of antibacterial agents. In this study, we present and characterize a (C^S)-cyclometallated Au(III) dithiocarbamate complex as a potential drug candidate.

**Methods and results:**

The Au(III) complex was found to be stable in the presence of effective biological reductants, and showed potent antibacterial and antibiofilm activity against a wide range of multidrug-resistant strains, particularly gram-positive strains, and gram-negative strains when used in combination with a permeabilizing antibiotic. No resistant mutants were detected after exposing bacterial cultures to strong selective pressure, indicating that the complex may have a low propensity for resistance development. Mechanistic studies indicate that the Au(III) complex exerts its antibacterial activity through a multimodal mechanism of action. Ultrastructural membrane damage and rapid bacterial uptake suggest direct interactions with the bacterial membrane, while transcriptomic analysis identified altered pathways related to energy metabolism and membrane stability including enzymes of the TCA cycle and fatty acid biosynthesis. Enzymatic studies further revealed a strong reversible inhibition of the bacterial thioredoxin reductase. Importantly, the Au(III) complex demonstrated low cytotoxicity at therapeutic concentrations in mammalian cell lines, and showed no acute *in vivo* toxicity in mice at the doses tested, with no signs of organ toxicity.

**Discussion:**

Overall, these findings highlight the potential of the Au(III)-dithiocarbamate scaffold as a basis for developing novel antimicrobial agents, given its potent antibacterial activity, synergy, redox stability, inability to produce resistant mutants, low toxicity to mammalian cells both *in vitro* and *in vivo*, and non-conventional mechanism of action.

## 1. Introduction

Bacterial antimicrobial resistance (AMR) has become a major health threat due to the emergence and spread of multidrug-resistant (MDR) bacteria. This has been fueled by the overuse and misuse of antimicrobial agents in both human and animal health, creating an avoidable selective pressure. A recent systematic review estimates that bacterial AMR was directly responsible for 1.27 million deaths in 2019 and contributed to an additional 3.68 million deaths, making it a significant worldwide concern and positioning it ahead of HIV and malaria in terms of magnitude (Murray et al., 2022). In response to this urgent public health crisis, the World Health Organization (WHO) published a list of priority pathogens in 2017 to focus efforts on antimicrobial discovery against these drug-resistant bacteria, particularly critical gram-negative species such as *Acinetobacter baumannii*, *Pseudomonas aeruginosa* and community-acquired *Salmonella* spp. (Tacconelli et al., 2018).

However, the clinical and preclinical antimicrobial pipeline falls short of meeting the need for effective drugs, with the majority of the clinical pipeline consisting of derivatives of known antibiotics, and less than 20% of the preclinical pipeline targeting the WHO’s critical gram-negative priority pathogens (World Health Organization, 2021). This underscores the need for the development of novel antibacterial agents, preferably from new chemical classes with new targets or non-traditional approaches, that are free from pre-existing cross-resistance (León-Buitimea et al., 2020; Iskandar et al., 2022).

In this context, metal-containing compounds are emerging as a versatile source for drug discovery due to the vast range of metals, ligand types, oxidation states, and geometries that offer endless possibilities for antibacterial applications (Frei et al., 2020). Among these, gold-based compounds in particular have received significant attention in recent years (Dominelli et al., 2018; Patanjali et al., 2018). Gold’s medicinal properties have been known since ancient times in Indian and Chinese cultures (Huaizhi and Yuantao, 2001; Subramaniyan Parimalam et al., 2021), and its antibacterial properties were first described by Robert Koch in the late 19^th^ century, who demonstrated the *in vitro* inhibitory effect of potassium dicyanidoaurate(I), K[Au(CN)_2_], against *Mycobacterium tuberculosis* (Benedek, 2004). Since then, numerous Au(I) and Au(III) complexes have been studied for their antibacterial properties (Ratia et al., 2022b), with the notable example of the FDA-approved antirheumatic Au(I) drug auranofin, recently repurposed as an antimicrobial drug under the trade name Ridaura^®^ (Cassetta et al., 2014; Liu et al., 2022). While auranofin and its Au(I) analogs are known to have potent antibacterial activity against several pathogenic gram-positive bacteria, including methicillin-resistant *Staphylococcus aureus* (MRSA), *Enterococcus faecalis*, and others like *M. tuberculosis* (Harbut et al., 2015; Thangamani et al., 2016; Marzo et al., 2018), their activity against gram-negative bacteria is limited (Fillat et al., 2011; Barreiro et al., 2012) with only a few exceptions (Özdemir et al., 2004; Frik et al., 2012).

Organometallic Au(III) complexes, although their antibacterial activities have been less studied compared to Au(I) complexes (Glišić and Djuran, 2014), attract particular attention as experimental anticancer drugs due to their close similarity to the cytotoxic agent cisplatin (Bertrand et al., 2018). Cyclometallated (C^N) Au(III) complexes are of particular interest because of their relatively high stability and their chemical plasticity. Parish et al. analyzed the antimicrobial activity of [AuCl_2_(damp)] and other cyclometallated analogs, observing a moderate broad-spectrum activity and selectivity toward gram-positive bacteria (Parish et al., 1996; Parish, 1999). More recently, Au(III) complexes with cyclometallated bidentate C^N scaffolds showed efficacy against *Bacillus subtillis* and *S. aureus* (Chakraborty et al., 2021), similar to Au(III) N-heterocyclic carbene complexes targeting MRSA and *Enterococcus faecium* (Büssing et al., 2021) and Au(III) bis(dithiolene) complexes against *S. aureus* (Le et al., 2022), but their efficacy against gram-negative strains is still insufficient. Our group recently reported the synthesis and characterization of a novel (C^S)-cyclometallated dichloro Au(III) complex **1** based on an *ortho*-substituted phosphinothioic amide which showed significant antibacterial activity against a broad spectrum of bacterial strains belonging to different gram-positive and gram-negative species. The complex demonstrated excellent synergistic activity with colistin against gram-negative MDR strains and was particularly effective against MRSA (Ratia et al., 2022a).

The mechanisms by which Au(III) complexes show antibacterial efficacy are still not fully understood (da Silva Maia et al., 2014). Impaired bacterial cell membrane integrity appears to play an important function in the activity of these compounds; recently, we observed that the (C^S)-cyclometallated Au(III) complex **1** can rapidly permeabilize the membrane, leading to the compound’s immediate uptake causing severe ultrastructural damage in MRSA, *A. baumannii* and *P. aeruginosa* (Ratia et al., 2022a), a finding that is consistent with the mode of action observed for some Au(I) complexes (Samanta et al., 2013). The outer membrane of gram-negative bacteria could also serve as a barrier, limiting the intracellular effect of Au(III) complexes. In terms of intracellular targets, gold compounds such as auranofin and other Au(I) complexes have shown to be strong inhibitors of the bacterial thioredoxin reductase (TrxR) (Harbut et al., 2015; Schmidt et al., 2017; Büssing et al., 2021). The reductive thioredoxin (Trx) enzyme is critical for maintaining redox homeostasis in gram-positive bacteria, while in most gram-negative species, it functions in parallel with the glutathione-glutaredoxin (GSH) system. As a result, targeting the Trx system in gram-positive bacteria lacking glutathione can lead to strong bactericidal effects, while gram-negative species are generally less sensitive to Trx system inhibition (Ren et al., 2019).

Modifying the ligands attached to the gold ion is a well-known strategy to modulate the biological properties of Au(III) complexes. Dithiocarbamate (dtc) ligands, which efficiently stabilize Au(III) cations (Nilakantan et al., 2016), have been widely used as ancillary ligands in the development of Au(III)-based metallodrugs (Ronconi et al., 2006). While early studies on the association of Au(III) with dtc ligands for antimicrobial purposes demonstrated inhibitory effects against *Streptococcus pneumoniae* and *Micrococcus luteus* (Criado et al., 1992) their potential as antimicrobial drugs and bacterial targets have remained poorly explored until recently (Soto et al., 2019; Ratia et al., 2022c).

**Figure 1:**
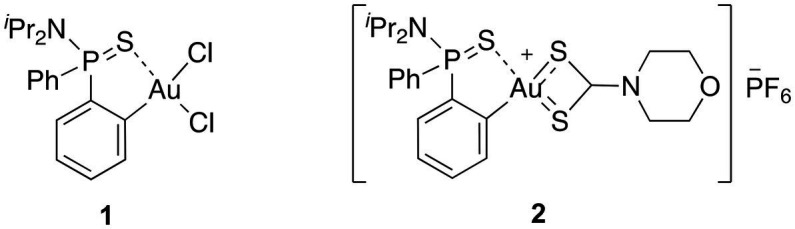
The molecular structure of the (C^S)-cyclometallated Au(III) complexes investigated in this study.

In this context, we envisioned that the coupling of a (C^S)-cyclometallated Au(III) complex with a dtc moiety could contribute to enhance both the stability of the complex and the antibacterial properties. With this in mind, we investigated the potential of a cationic Au(III) complex **2**, in which the Au(III) center is stabilized by a *ortho*-thiophosphinamide ligand and a dtc ligand, [Au^III^(C^S)(R_2_NCS_2_)]^+^ (Figure 1) as a new chemical drug to target MDR bacteria. By synthesizing and characterizing complex **2**, we discovered that it exhibits strong activity against gram-positive MDR isolates, and in combination with a permeabilizing antibiotic, also showed activity against gram-negative strains. Our study suggests that complex **2** acts through a multi-targeted mechanism of action, involving both the inhibition of the bacterial TrxR and membrane permeabilization. Furthermore, complex **2** exhibited low toxicity *in vitro* and *in vivo*, and no resistant mutants were produced, indicating its potential as an effective and safe antimicrobial agent.

## 2. Materials and methods

### 2.1. Synthesis of [Au(dppta)(mrdtc)][PF_6_] complex 2

To a solution of [Au(dppta)Cl_2_] complex **1** (117 mg, 0.20 mmol) in MeOH (6 mL), sodium morpholine-4-carbodithioate was added (0.20 mmol). The reaction mixture was stirred at rt for 12 h and then aqueous saturated potassium hexafluorophosphate was added and the mixture was stirred for 15 min. After partial evaporation of the methanol, the resulting solid was filtered and the residue was washed with water and diethyl ether to afford desired [Au(dppta)(mrdtc)][PF_6_] complex **2** (63% yield).

### 2.2. Stability of [Au(dppta)(mrdtc)][PF_6_] complex 2

A solution of compound **2** in CD_3_CN (0.25 mL) was mixed with 0.25 mL of ISOsensitest culture broth prepared in D_2_O and the ^31^P NMR spectra of was monitored at 37°C over three days. The complex remained unaltered over this period.

Solutions of compound **2** in CD_3_CN (0.25 mL) were mixed with solutions containing equimolar amounts of either GSH or AsAc in 0.25 mL of D_2_O. The ^31^P NMR spectrum clearly indicated that the gold complex maintained its chemical integrity under these conditions (Pantelić et al., 2017).

### 2.3. Bacterial strains, growth conditions, and reagents

The bacterial clinical isolates used in this study, including their source, general characteristics, and resistance phenotypes, are listed in Tables 1, 2. Reference strains *S. aureus* ATCC 29213, *S. pneumoniae* ATCC 49619, *P. aeruginosa* ATCC 27853, *E. coli* ATCC 25922 and *A. baumannii* ATCC 19606 were included as controls. Bacterial species identification was confirmed by Matrix-assisted laser desorption ionization-time of flight (MALDI-TOF) mass spectrometry. Isolates were cultured on Columbia Sheep Blood Agar (Becton Dickinson, Heidelberg, Germany) and incubated at 37°C for 18 h, except for *S. pneumoniae* strains, which were cultured with 5% CO_2_, *B. cepacia* complex strains, which were incubated for 48 h, and *H. influenzae* isolates, which were grown in Chocolate Blood Agar containing Iso-VitaleX (Becton Dickinson, Heidelberg, Germany) at 37°C with 5% CO_2_. The antibiotics used in the experiments were auranofin (Cayman Chemical), amikacin (AMK, AlfaAesar), ampicillin (AMP, SigmaAldrich), colistin (CST, MPBiomedicals), ciprofloxacin (CIP, SigmaAldrich), daptomycin (DAP, SigmaAldrich), gentamicin (GEN, SigmaAldrich), linezolid (LZD, SigmaAldrich), and rifampicin (RIF, SigmaAldrich). Stock solutions of complex **2** and auranofin were prepared in 100% dimethyl sulfoxide (DMSO, Sigma-Aldrich, Darmstadt, Germany) at 10 mM. Stock solutions of commercial antibiotics were prepared according to the manufacturer’s instructions.

**Table 1:** Median MIC values for complex 2 and the reference drug auranofin against a panel of clinical gram-positive drug-resistant isolates and reference strains.

Strain ID	Species	Characteristics	Resistance	Source	MIC (μM)	
					2	Auranofin
162-065-705	*S. aureus*	MRSA	CIP, LVX, CLI, ERI, PEN	Respiratory isolate (*CF*). Hospital Clínic (Barcelona, Spain)	0.15	0.18
163501-000	*S. aureus*	MRSA	CIP, CLI, ERI, LVX	Respiratory isolate (*CF*). Hospital Clínic (Barcelona, Spain)	0.30	0.18
162071-210	*S. aureus*	MRSA	CIP, CLI, ERI, LVX	Respiratory isolate (*CF*). Hospital Clínic (Barcelona, Spain)	0.07	0.18
162058-967	*S. aureus*	MSSA	AMK, TET, CIP, CHL, ERI, VAN	Respiratory isolate (*CF*). Hospital del Mar (Barcelona, Spain)	0.15	0.18
ATCC^®^ 29,213	*S. aureus*	MSSA	–	Wound. Reference stain American Type Culture Collection	0.15	0.18
FG22014	*S. epidermidis*	MSSE	PEN, CTX, CRO, CIP, CHL, ERI	Wound. Hospital Clínic (Barcelona, Spain)	0.15	0.18
FG03015	*S. epidermidis*	MRSE	OXA, PEN, CTX, CIP, ERI	Wound. Hospital Clínic (Barcelona, Spain)	0.15	0.18
FG14013	*S. epidermidis*	MRSE	OXA, PEN, CTX, GEN, CIP, CHL, ERI	Wound. Hospital Clínic (Barcelona, Spain)	0.15	0.18
345	*S. pneumoniae*		PEN	Respiratory isolate. Vall d’Hebron Hospital (Barcelona, Spain)	2.44	0.74
14	*S. pneumoniae*		PEN	Blood. Vall d’Hebron Hospital (Barcelona, Spain)	2.44	1.47
ATCC^®^ 49,619	*S. pneumoniae*		–	Sputum. Reference stain American Type Culture Collection	1.22	0.74

**Table 2:** Median MIC values for complex 2 and the reference drug auranofin against a panel of clinical gram-negative drug-resistant isolates and reference strains.

Strain ID	Species	Characteristics	Resistance	Source	MIC (μM)	
					2	Auranofin
9510-524	*S. maltophilia*		CAZ, SXT	Respiratory isolate (*CF*). Hospital Clínic (Barcelona, Spain)	4.87	23.58
895	*S. maltophilia*		LVX, CAZ, CHL, SXT	Respiratory isolate (*CF*). Hospital Clínic (Barcelona, Spain)	9.75	23.58
166097-953	*P. aeruginosa*		TOB, GEN, CIP, AZT, LVX, IMP	Respiratory isolate (*CF*). Hospital Clínic (Barcelona, Spain)	9.75	94.33
30302995-242	*P. aeruginosa*		GEN, TOB, TZP, CIP, IMP, MEM, AZT, LVX, IMP, DOR	Respiratory isolate (*CF*). Hospital del Mar (Barcelona, Spain)	9.75	>200
ATCC^®^ 27,853	*P. aeruginosa*	-	-	Blood. Reference stain American Type Culture Collection	9.75	>200
E4	*E. coli*	CTX-M − 15, TEM-1, SHV-12	CHL, GEN, NAL, TET, SXT, RIF	Blood. Manhiça District Hospital (Manhiça, Mozambique)	9.75	>200
E8	*E. coli*	CTX-M − 37, TEM-1, OXA-1	CHL, GEN, CIP, NAL, TET, SXT, RIF	Urine. Manhiça District Hospital (Manhiça, Mozambique)	4.87	>200
E11	*E. coli*	CTX-M − 15, TEM-1, OXA-1	CHL, GEN, CIP, NAL, TET, SXT, RIF	Blood. Manhiça District Hospital (Manhiça, Mozambique)	4.87	>200
ATCC^®^ 25,922	*E. coli*		-	Reference stain American Type Culture Collection	9.75	94.33
3,670	*H. influenzae*		SXT	Respiratory isolate (*CF*). Bellivitge Hospital University (Barcelona, Spain)	0.61	>200
3,150	*H. influenzae*		SXT	Respiratory isolate (*CF*). Bellivitge Hospital University (Barcelona, Spain)	0.61	>200
AbCr17	*A. baumannii*	PanR	AMC, CIP, LVX, MOX, DOX, SXT, TOB, AMK, CST, MEM, RIF, AMP, GEN	Cerebrospinal fluid. Virgen del Rocío University Hospital (Seville, Spain)	4.87	94.33
Ab177	*A. baumannii*	OXA-58	AMC, CIP, LVX, MOX, DOX, SXT, TOB, AMK, MEM, RIF, AMP, GEN	Blood. GEIH-REIPI-Ab 2010 Study	9.75	47.16
Ab210	*A. baumannii*	OXA-23	AMC, CIP, LVX, MOX, DOX, SXT, AMK, CST, MEM, RIF, AMP, GEN	Absominal drain fluid. GEIH-REIPI-Ab 2010 Study	9.75	94.33
ATCC^®^ 19,606	*A. baumannii*	-	-	Urine. Reference stain American Type Culture Collection	4.87	47.16
HCB0093	*K. pneumoniae*	TEM-1, CTX-M,	AMC, IMI, TZP, CAZ, CIP, SXT	Respiratory isolate. Hospital Clínic (Barcelona, Spain)	19.50	>200
HCB265	*K. pneumoniae*	SHV-1, TEM-1, CTX-M, tetA	AMC, SXT, CAZ, CEF, CIP, AZT, CHL	Respiratory isolate. Hospital Clínic (Barcelona, Spain)	19.50	>200
HUB725	*K. pneumoniae*	TEM-1, CTX-M	AMC, SXT, CAZ, CIP, CST, AZT, FOS CEF, CTX	Urine. Bellivitge Hospital University (Barcelona, Spain)	19.50	>200
HCB310	*K. aerogenes*		AMC, SXT, IMI, MEM, DOX, CTX, CAZ, CIP, FOF	Respiratory isolate. Hospital Clínic (Barcelona, Spain)	19.50	>200
HMT330	*E. cloacae*		SXT, DOX, CAZ, FOS	Urine. MútuaTerrassa University Hospital (Terrassa, Spain)	19.50	>200
SHI1	*S. sonnei*		NAL, AMP, CIP, SXT, AZT	Fecal. Vall d’Hebron Hospital (Barcelona, Spain)	9.75	23.58
SHI3	*S. sonnei*		NAL, AMP, CIP, SXT, AZT	Fecal. Vall d’Hebron Hospital (Barcelona, Spain)	9.75	23.58
LSP 23/12	*S. enteritidis*		CIP, CST, AMP	Fecal. Asturias Central University Hospital (Oviedo, Spain)	19.50	>200
3	*S. enterica*		CIP, DOX, AMP, SXT	Fecal. Asturias Central University Hospital (Oviedo, Spain)	4.87	>200
41	*B. cepacia* complex		CHL	Respiratory isolate (*CF*). Ramon y Cajal Hospital (Madrid, Spain)	9.75	>200
42	*B. cepacia* complex		CHL, DOX, LEV	Respiratory isolate (*CF*). Ramon y Cajal Hospital (Madrid, Spain)	9.75	>200

### 2.4. Minimum inhibitory concentration determination

The minimum inhibitory concentrations (MICs) of complex **2** against the listed strains were determined in triplicate by the broth microdilution method recommended by the Clinical and Laboratory Standards Institute (CLSI) (Dolinsky, 2021) in 96-well round-bottom microtiter plates. Auranofin, a reference Au(I) complex, was included as a comparator antimicrobial agent. Assays were performed in ISO-Sensitest broth (Oxoid, Madrid, Spain), supplemented with 5% (v/v) lysed horse blood for *S. pneumoniae* strains and in *Haemophilus* Test Medium (HTM) for *H. influenzae* strains. The plates were incubated at 37°C (with 5% CO_2_ for *H. influenzae* and *S. pneumoniae* isolates) and were read after 18 h (24 h for *B. cepacia* complex strains) for the absence of turbidity. MIC values were defined as the lowest concentration of the compound that inhibited visible growth.

### 2.5. Time-dependent killing assays

We evaluated the bactericidal activity of **2** against MRSA 1620579-000 and *A. baumannii* Cr17 in a time-dependent killing assay. Macrodilution series of complex **2** were prepared in ISO-Sensitest broth with concentrations correlating to their respective MICs (0.5x, 1x, 2x and 4x MIC). The tubes were inoculated to reach a bacterial density of 5.10^5^ CFU/mL per tube and incubated at 37°C with shaking at 180 rpm. Viable bacterial counts were determined by taking aliquots at specific time points (0, 2, 4, 8, 24, and 48 h) and plating for colony counting after 18 h at 37°C.

To assess the combination of complex **2** with CST and GEN in time-dependent killing assays, each tube of ISO-Sensitest broth was supplemented with either complex **2** alone or in combination with the antibiotic, at concentrations ranging from 0.03x MIC to 2x MIC for both drugs, following the same scheme as described above. Synergy was defined as a ≥ 2 log decrease in viability (CFU/mL) between the combination and the most active agent alone, as well as with the starting inoculum.

### 2.6. Transmission electron microscopy analysis

Bacterial ultrastructural analysis after treatment with complex **2** alone or in combination with CST and GEN was carried out by transmission electron microscopy (TEM) as previously described (Ratia et al., 2022a). Exponential cultures of MRSA 163501-000, *P. aeruginosa* 953 and *A. baumannii* AbCr17 were exposed to minimum inhibitory concentrations of complex **2**, CST, GEN and their combination for 30 min at 37°C prior to analysis.

### 2.7. Quantification of complex 2 bacterial uptake

MRSA 163501-000 and *A. baumannii* AbCr17 cultures were harvested to reach a 10^10^ CFU/mL cell density and treated after with 36.5 μM of complex **2**. Aliquots at 0, 5, and 8 min timepoints were digested with HNO_3_, H_2_O_2_, and HCl at 60°C and subsequently diluted in an HCl-thiourea solution. Parallel to lysis, samples were also plated for CFU determination. Untreated bacteria (PBS) were used as a control. The presence of Au in the samples was detected through Inductively coupled plasma-mass spectrometry (ICP-MS) in an Agilent 7500ce equipment under standard conditions.

### 2.8. Antibiofilm activity

The antibiofilm activity of complex **2** was evaluated against a subset of biofilm-forming strains selected from the initial panel. Both the minimum biofilm inhibitory concentration (MBIC) and minimum biofilm eradication concentration (MBEC) were determined. For *P. aeruginosa* and *A. baumannii*, LB medium supplemented with 0.25% glucose was used to promote biofilm formation, while TSB was used for the *B. cepacia* complex, *S. aureus*, *S. epidermidis*, *K. pneumoniae*, and *S. maltophilia*. Additionally, TSB diluted 1/20 was used for *S. enterica* strains. *P. aeruginosa* and *B. cepacia* complex biofilms were formed by immersing pegs of a modified polystyrene microtiter lid (Nunc TSP System, Nunc, Rockslide, Denmark).

The MBIC of **2** was determined by a modification of the broth microdilution method as described previously (Ratia et al., 2022a). Biofilm biomass was quantified by solubilizing the biofilm-attached dye in 33% v/v glacial acetic acid and measuring absorbance at 580 nm using a microplate spectrophotometer (EPOCH, Biotek, Santa Clara, CA, United States). The MBIC was defined as the minimal concentration of the compound that resulted in a three-fold decrease in absorbance compared to the growth control values. All experiments were carried out in triplicate.

To determine the MBEC, bacterial suspensions were prepared in the corresponding media from overnight cultures, adjusted to 5.10^6^ CFU/well in 96-well flat-bottom polysterene plates and incubated at 37°C for 48 h. After incubation, biofilms were washed to remove non-adherent cells and treated with serial two-fold dilutions of **2** at 37°C for 24 h. Following treatment, the medium was gently removed from each well, replaced with a 16 μg/mL solution of AlamarBlue and incubated 3 h at 37°C (Van den Driessche et al., 2014). Fluorescence generated by metabolically active bacteria was measured at 530/590 nm using a Tecan microplate reader (Tecan Infinite M2000PRO, Zurich, Switzerland). The MBEC was defined as the minimal concentration of complex **2** that reduced bacterial viability by more than 95% compared to the untreated control. All experiments were carried out in triplicate.

### 2.9. Checkerboard assay

Two-dimensional checkerboard assays were performed in ISO-Sensitest broth to determine the interaction of complex **2** with clinical antibiotics AMK, AMP, CST, CIP. GEN, LZD and RIF. The setup of each assay plate evaluated 2-fold dilutions of **2** with 2-fold dilutions of the antibiotic. Inoculum size, culture media and incubation conditions were the same as described above for the MIC microdilution method. Results are read after 18 h by visual examination. The fractional inhibitory concentration index (FICI) was calculated considering the following formula: FICI = FIC A + FIC B, where FIC A is the MIC of drug A in combination/MIC of drug A alone, and FIC B is the MIC of drug B in combination/MIC of drug B alone. Synergy occurs when the combined activity of two antimicrobials is greater than the sum of their individual activities and is defined by a FICI value of ≤0.5. Interactions with FICI values ranging from 0.5 to ≤1 are classified as additive, meaning that the cumulative antimicrobial effect is simply the sum total of the two antimicrobials acting together. An interaction is considered indifferent if the FICI is >1–4, and it is classified as antagonistic if the FICI is ≥4 (Saiman, 2007). Results were validated in triplicate.

### 2.10. Assessment of resistance development

We performed sequential culturing of MRSA 163501-000 and *A. baumannii* AbCr17 strains in the presence of subinhibitory levels of complex **2**, with DAP and CST as antibiotic controls. Bacterial cultures grown to OD = 1 were diluted 1:100 with complex **2** concentrations ranging from 0.25x MIC to 8x MIC. At 24-h intervals, the highest concentration that allowed bacterial growth was used as the inoculum for a new macrodilution series of **2**, with an increased concentration range if necessary. This serial passage was repeated for 30 days, with aliquots taken from each passage to determine changes in the initial MIC via broth microdilution.

### 2.11. Sample preparation for RNA sequencing

To investigate the effect of complex **2** on gene expression in *E. coli* ATCC 25922, liquid cultures were prepared in biological triplicate with or without complex **2** at 0.5x MIC. Once cultures reached mid-log phase with an OD600 of 0.5, cells were pelleted and treated with RNA protect. Total RNA was extracted by resuspending pellets in TE buffer containing 1 mg/mL lysozyme (Qiagen) and incubated for 5 min, followed by purification using the RNAeasy mini kit (Qiagen) and treated with the DNA-free™ kit (Ambion). RNA samples were quantified using a Qubit 2.0 fluorometer (Invitrogen) and assessed for quality using a Bioanalyzer (Agilent Technologies). Samples with an RNA integrity number greater than 7 were included in subsequent analyses.

### 2.12. RNA sequencing and analysis

To prepare samples for RNA sequencing (RNA-seq), total RNA was depleted of rRNA using the NEBNext^®^ rRNA depletion Kit (NewEngland Biolabs) prior to library construction. The NEBNext Ultra directional RNA library prep kit (NewEngland Biolabs) was used to generate cDNA libraries from 2–5 ng of the prepared samples. The quality of the libraries was assessed using Agilent DNA chips to confirm size distribution and the absence of small adapters. The libraries were then sequenced on an Illumina HiSeq2500 using a paired-end (2 × 75 bp reads) TopHat + Cufflinks protocol, producing 10 million reads per sample (Trapnell et al., 2012). For mapping reads and performing differential expression analysis, the latest version of the reference genome was used (*E. coli*).^1^ The TopHat mapper was used to map the treated and untreated samples against the reference genome. Differential expression analysis was carried out using the Cufflinks package (Trapnell et al., 2012). Firstly, the Cufflinks tool was used to assemble a transcriptome in each case. Then, Cuffdiff was used to quantify and test the differential expression between treated and untreated samples. To perform Gene Ontology (GO) enrichment analysis (Huntley et al., 2015), all transcripts were tested using the R GOseq package (Young et al., 2010) using the Wallenious distribution method. Significance thresholds were set at FDR < 0.05. Finally, metabolic pathway enrichment analysis based on Kyoto Encyclopedia of Genes and Genomes (KEGG) maps (Kotera et al., 2012) was performed using the GOseq application.

### 2.13. Production and purification of *Escherichia coli* TrxR and Trx

Human TrxR1 was expressed and purified as previously described (Cheng and Arnér, 2017). Following the already described approaches, DNA cassettes with sequences encoding *E. coli* TrxR (GenBank: PSY25180.1, residues 1–321), and Trx (GenBank: EDV64981.1, residues 1–109) were here synthesized by Integrated DNA Technologies, Inc. The individual open reading frames were subsequently subcloned into the inhouse pD441a plasmid to generate fusion proteins of His6-SUMO-EcTrxR and His6-SUMO-EcTrx, respectively (Cheng and Arnér, 2017). The plasmids were subsequently transformed into BL21(DE3) for protein production (Supplementary material S1).

The recombinant *E. coli* TrxR and Trx were expressed and purified essentially as described previously (Cheng and Arnér, 2017). Briefly, 20 mL of overnight cultures of transformed bacteria were inoculated into 1-liter terrific broth (TB) medium containing 50 μg/mL kanamycin in a 5-liter bottle placed on a shaking incubator at 37°C. Three hours after inoculation, temperature was lowered to 25°C with 0.5 mM IPTG added to induce protein expression. The bacteria were subsequently harvested next day by centrifugation, suspended in IMAC binding buffer (50 mM Tris–HCl, 500 mM NaCl, 10 mM imidazole, 10% glycerol, pH 7.5) and lysed by sonication. The soluble fraction was recovered by centrifugation and applied onto a HisTrapTMHP column (5 mL) equipped on an ÄKTA explorer FPLC system (Cytiva Life Sciences). The fusion protein was eluted with elusion buffer (IMAC binding buffer with additional 200 mM imidazole) and treated with inhouse produced His-tagged SUMO protease ULP1 (1%) overnight in a dialyzing bag soaked in 5-liter low salt buffer (50 mM Tris–HCl, pH 7.5). The mixture was subsequently re-applied onto the HisTrapTMHP column to separate non-tagged target protein from its N-terminal His-tagged fusion partner as well as from the His-tagged ULP1. The target proteins were finally concentrated, buffer exchanged (50 mM Tris–HCl, pH 7.5, with 2 mM EDTA, and 20% glycerol), and stored in −20°C freezer until further analyses. Purity of the enzymes were greater than 95% as assessed by SDS-PAGE.

### 2.14. Inhibition of bacterial thioredoxin reductase and human TrxR1

We assessed the dose-dependent inhibition of purified recombinant *E. coli* TrxR and human TrxR1 (HsTrxR1) using a modified DTNB reduction activity assay protocol (Lu et al., 2013). For the *E. coli* TrxR, graded concentrations of complexes **1, 2** and auranofin (0.01–100 μM) were pre-incubated for 30 min with a 2x TrxR-Trx1 solution (without NADPH) in TE buffer (Tris–HCl 50 mM, EDTA 1 mM, pH 7.5). Control samples contained DMSO instead of inhibitor. After incubation, the reaction was started in 96-well plates by adding an equal volume of NADPH and DTNB as end substrate, to reach a final assay concentration of 2.5 μM *E. coli* Trx 1, 25 nM *E. coli* TrxR, 0.25 mM NADPH and 1 mM DTNB. For HsTrxR1, same graded concentrations of inhibitors were pre-incubated for 30 min with a 2x HsTrxR1 and NADPH solution in TE buffer. After incubation, the reaction was started in 96-well plates by adding an equal volume of DTNB as end substrate, to reach a final assay concentration of 25 nM HsTrxR1, 0.25 mM NADPH and 1 mM DTNB. For both enzymes, absorbance at 412 nm was monitored and TrxR activity was calculated as the slopes (increase in absorbance per second) during the initial linear phase of the reaction. The fractional activities were calculated relative to the control samples. Data was plotted as triplicate means ±SD and fitted on a dose–response curve to calculate IC_50_ values.

For time-dependent inhibition assays, we measured the *E. coli* TrxR activity in the presence of a constant concentration of the Au complexes (equal to the IC_50_) at different incubation times (2, 5, 10, 20, 30, 60, 90 and 120 min). Reactions and reagent concentrations were the same as in the DTNB assay described above.

### 2.15. Molecular docking

All enzymes were prepared using the Protein Preparation Wizard and Propka from the Schrödinger package. TrxR was modeled using available NMR structures of the active conformation from *E. coli* (pdb code: 1F6M), obtained from a complex of thioredoxin and thioredoxin reductase. Complex **2** was parameterized employing quantum mechanics (QM) calculations, using the CP2K software (Vandevondele et al., 2005; Hutter et al., 2014). Initial conformation of **2** was built manually using standard computational tools and subsequently energy minimized at the density functional theory (DFT) level using the CP2K software. Complex **2** was initially prepared using the Ligand Preparation Wizard LigPrep tool from Schrödinger. Docking simulations were carried out against the 20 NMR Thioredoxin [Trx-(SH)2] structures using the GLIDE module from Schrödinger. Quantum Mechanics/Molecular Mechanics (QM/MM) calculations were carried out starting from rigid GLIDE docking conformations to perform a final optimization/refinement of the **2**-bound conformation. Both, **2** and nucleophile residue Cys32, were described using PBE level of DFT through the CP2K software (Vandevondele et al., 2005; Hutter et al., 2014). The DZVP-MOLOPT-SR-GTH pseudo-potential was used to describe Au; and the DZVP-MOLOPT-GTH pseudo-potential was used to describe the electronic properties of C, N, O, S, P, and H. The rest of the system was described at the classical (MM) level using the amber ff14SB force field for proteins (Maier et al., 2015) and the TIP3P water model (Jorgensen et al., 1983).

### 2.16. *In vitro* toxicity in mammalian cells

Cytotoxicity in terms of antiproliferative effect was assessed by 3-(4,5-dimethylthiazol-2-yl)-2,5-diphenyltetrazolium bromide (MTT) colorimetric assay. The study included tumoral liver HepG2 (HB-8065) cells and non-tumoral human liver THLE-2 cells. Cells were seeded at an initial concentration of 1 × 10^4^ cells/well in 200 μL of Eagles’s minimum essential medium (MEM) and incubated at 37°C in 5% CO_2_. Graded concentrations of compounds or DMSO (in control wells) were added to the cells, and the plates were incubated for 72 h at 37°C in 5% CO_2_. After this time, MTT solution was prepared at 5 mg/mL in 1x PBS and then diluted to 0.5 mg/mL in MEM without phenol red. The sample solution in the wells was flicked off, and 100 μL of MTT dye was added to each well. The plates were gently shaken and incubated for 3 h at 37°C in 5% CO_2_. The supernatant was removed, and 100 μL of 100% DMSO was added. The plates were gently shaken to solubilize the formed formazan. The absorbance was measured at 570 nm using a multireader VictorTM.

### 2.17. *In vivo* acute toxicity in mice

Female CD1 mice (ICR) aged 7–8 weeks (*n* = 5/group) were obtained from Charles River Laboratories (Écully, France). All experimental procedures were approved by the Animal Experimentation Ethical Committee of the University of Barcelona (CEEA 82/16). Complex **2** was administered intravenously as a single dose (2 mg/kg or 5 mg/kg) as treatment, while the control groups were vehicle-treated (0.1% DMSO) or untreated. Mice were monitored for 14 days after treatment for signs of toxicity such as weight reduction, bristly coat, reduced mobility, and ocular epiphora. A reduction below 80% of the initial mice weight was considered an endpoint criterion. At the end of the experiment, all animals were anesthetized, sacrificed by cervical dislocation, and vital organs were excised, weighed, and examined macroscopically for signs of toxicity. The results were expressed as the organ weight (OW)/body weight (BW) coefficient.

## 3. Results and discussion

### 3.1. Synthesis and stability of complex 2

The target (C^S)-cyclometallated Au(III) complex **2** was synthesized using established procedures (Ratia et al., 2022a,c). Briefly, treatment of a methanolic solution of [Au(dppta)Cl_2_] complex **1** (dppta = *ortho*-*N,N*-diisopropyl-*P,P*-diphenylphosphinothioic amide) (Ratia et al., 2022a) with sodium morpholine-4-carbodithioate, followed by the addition of aqueous potassium hexafluorophosphate afforded the desired [Au(dppta)(mrdtc)][PF_6_] complex **2** (mrdtc = morpholine-4-dithiocarbamate) in 63% yield. The structure of complex **2** was confirmed using various analytical techniques, including 1D and 2D NMR, HRMS, IR, and UV.

**Figure 2:**
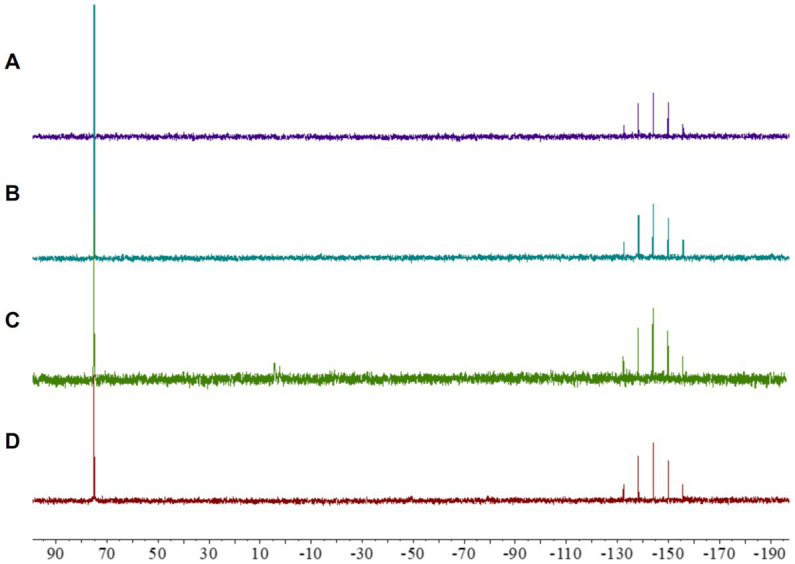
^31^P NMR spectra of [Au(dppta)(mrdtc)][PF_6_]complex 2: **(A)** in CD_3_CN; **(B)** in a 0.25 mL CD_3_CN/0.25 mL of ISO-Sensitest solution; **(C)** in a 0.25 mL CD_3_CN/0.25 mL aqueous ascorbic acid (1.0 equiv) solution; **(D)** in a 0.25 mL CD_3_CN/0.25 mL GSH (1.0 equiv) solution.

Initial analyses of complex **2** showed a remarkable lack of fragmentation in the mass spectra, suggesting a notable stability of the compound (Fan et al., 2003). Given that maintaining the integrity of the metallocentre is crucial to observe any biological activity (Parish, 1999), we further investigated the stability of the cyclometallated Au(III) complex **2** under the conditions used for biological evaluations. To this end, a solution of complex **2** in CD_3_CN was treated with ISO-Sensitest media and incubated at 37°C during 24 h, and the subsequent ^31^P NMR revealed that the complex maintained its structural integrity under those conditions (Figure 2).

Despite the promising biological activity of several cyclometallated Au(III) complexes, their application in pharmacology has been severely hampered by a tendency to undergo reduction in the presence of biologically relevant molecules (Yeo et al., 2018). Many studies have shown that reducing substances, such as ascorbic acid and thiol-containing amino acids, cause rapid reduction of Au(III) to Au(I) or Au(0) (Pantelić et al., 2017), resulting in the accumulation of oxidatively modified proteins in mammalian tissues, including the liver, heart, skeletal muscle, kidney, and brain regions (Ba et al., 2011). Therefore, we investigated the possibility that [Au(dppta)(mrdtc)][PF_6_] complex **2** could be reduced in cells using ^31^P NMR. As presented in Figure 2, when a solution of complex **2** in CD_3_CN was treated with an aqueous solution of ascorbic acid, the ^31^P NMR spectrum indicated that the Au(III) metallocentre remained structurally intact. Similarly, incubating complex **2** in CD_3_CN with reduced glutathione for 24 h, the ^31^P NMR spectrum again showed that the complex remained unaltered (Figure 2). These results suggest that direct chemical oxidative reactions of the Au(III) complex **2** with cellularly relevant reductants may be limited.

### 3.2. Antibacterial activity of complex 2

Complex **2** exhibited potent antibacterial activity against a variety of gram-positive isolates (Table 1), including methicillin-resistant *S. aureus* (MRSA) and methicillin-susceptible *S. aureus* (MSSA) (MIC values 0.07–0.30 μM), *S. epidermidis* (MIC 0.15 μM) and *S. pneumoniae* (MIC values 1.22–2.44 μM). The differences on MIC between *Staphylococcus* and *Streptococcus* strains could be due to the capsule present in the last ones, avoiding the penetration of the complex **2** to the cell. No significant differences in activity were observed among isolates with varying antimicrobial resistance profiles from the same species. In addition, the concentrations of complex **2** needed to inhibit gram-positive growth were similar to those obtained with auranofin (Table 1). Although selectivity for gram-positive strains at low concentration ranges is common among Au(III) complexes (Büssing et al., 2021; Chakraborty et al., 2021), here we found that complex **2** exhibited significantly stronger activity compared to the MICs of our previously synthesized [Au(dppta)Cl_2_] complex **1** against the same MRSA, MSSA and *S. epidermidis* strains (Ratia et al., 2022a). Thus, complex **2** has the lowest reported MIC values of an Au(III) complex against gram-positive pathogens to date, and has similar activity than the Au(I) complex auranofin against MRSA, MSSA and *S. epidermidis*.

Complex **2** exhibited less activity against gram-negative compared to gram-positive bacteria, with certain inter-species variability (Table 2). We observed a notable inhibitory effect against *H. influenzae* strains (MIC 0.61 μM) and, to a lesser extent, against *S. maltophilia, E. coli, S. enterica* and *A. baumannii* (MIC 4.87 μM). Interestingly, Büssing et al. (2021) also reported a stronger activity of an Au(III)-NHC carbene complex against *A. baumannii* compared to other Gram-negative strains. The MIC values were moderate ranging from 9.75 to 19.50 μM for *P. aeruginosa,* certain *E. coli*, *K. pneumoniae, K. aerogenes, E. cloacae, S. sonnei, S. enteritidis* and *B. cepacia* complex strains. These differences could be due to the different composition on carbohydrates and lipids of the outer membranes from the different gram-negative species. Importantly, complex **2** exhibited considerably higher antibacterial efficacy than auranofin, which was only minimally active against gram-negative strains (Table 2). This is likely due to the poor penetration of auranofin through the outer membrane of gram-negative bacteria (Thangamani et al., 2016). As observed for gram-positive strains, the efficacy of complex **2** did not vary against drug-resistant and drug-sensitive strains of the same species, suggesting that cross-resistance with clinical antibiotics is unlikely to occur.

**Figure 3:**
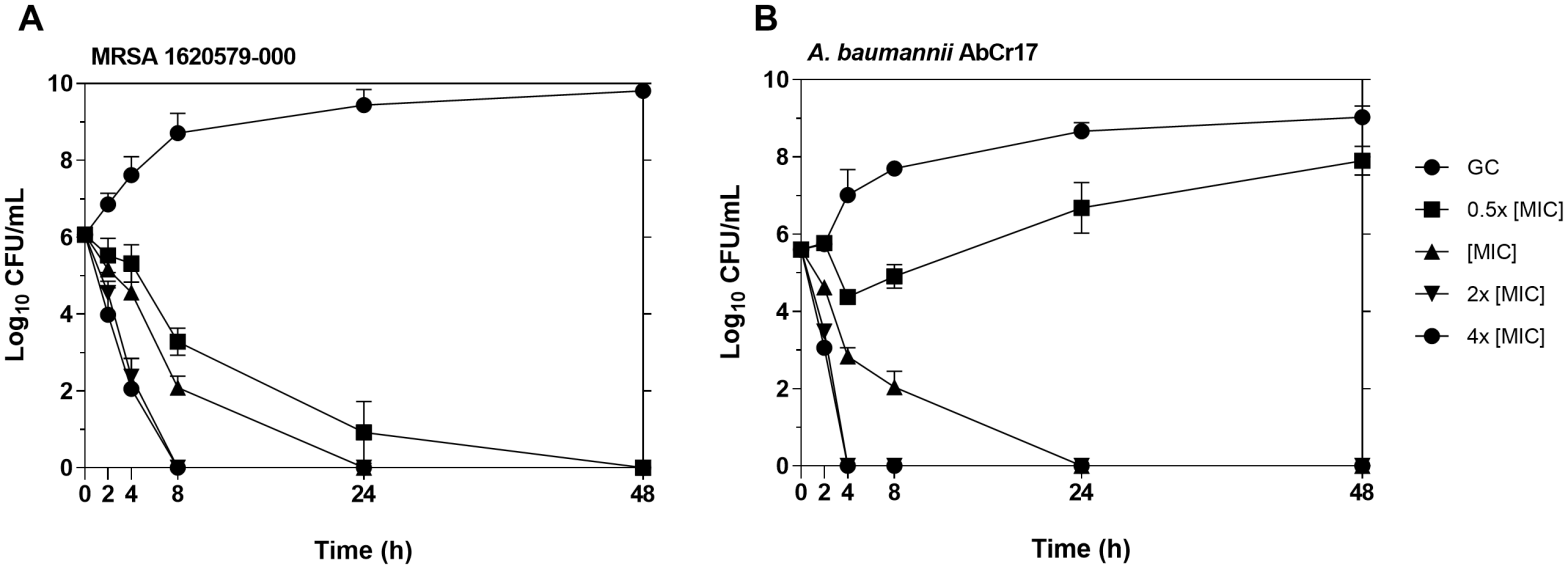
Time-kill kinetics of complex 2 against MRSA 163501-000 **(A)** (MIC = 0.30 μM) and *A. baumannii* AbCr17 **(B)** (MIC = 4.87 μM). The concentrations of complex 2 used for the experiment were 0.5x, 1x, 2x and 4x MIC. Bacterial viability (CFU/mL) was obtained at given time points. GC, growth control.

To better understand the antibacterial activity of Au(III)-dtc complex **2**, we conducted time-dependent killing assays against MRSA 163501-000 and *A. baumannii* AbCr17 as representatives of gram-positive and gram-negative strains and due to the clinical relevance of both strains, as AbCr17 is a colistin-resistant isolate that is considered PDR, while *S. aureus* ID163501-000 is a strong biofilm-forming MDR isolate from a cystic fibrosis patient. Noteworthy, in both gram-positive and gram-negative species, the complex **2** has a bactericidal effect at the MIC concentration after 24 h of incubation. In MRSA (Figure 3A), complex **2** showed a ≥ 5 log CFU/mL reduction in 8 h at 2x MIC (0.60 μM) and in 24 h at 4x MIC (1.2 μM), respectively. For *A. baumannii* (Figure 3B), the viable cell counts quickly decreased by ≥5 log CFU/mL within 4 h at 2x MIC (9.75 μM) and 4x MIC (19.48 μM), also achieving a bactericidal effect in 24 h at MIC. The differences on MIC concentration observed among MRSA and AbCr17 to obtain the same effect could be due to the presence of the outer membrane in the last one. Thus, complex **2** can effectively kill both MRSA and *A. baumannii* in a short timeframe, suggesting a time- and dose-dependent bactericidal activity with no signs of regrowth after 48 h.

Overall, complex **2** selectively targets gram-positive strains at low micromolar concentrations. It also demonstrates remarkable efficacy against gram-negative pathogenic *H. influenzae, E. coli* and *A. baumannii* strains, exceeding the activity of the reference Au(I) drug, auranofin. Complex **2** exhibits a bactericidal effect at concentrations above the MIC, and its activity is consistent across isolates, regardless of resistance phenotype or strain origin, confirming its effectiveness against MDR pathogenic bacteria.

### 3.3. Ultrastructural analysis of complex 2-treated bacteria

**Figure 4:**
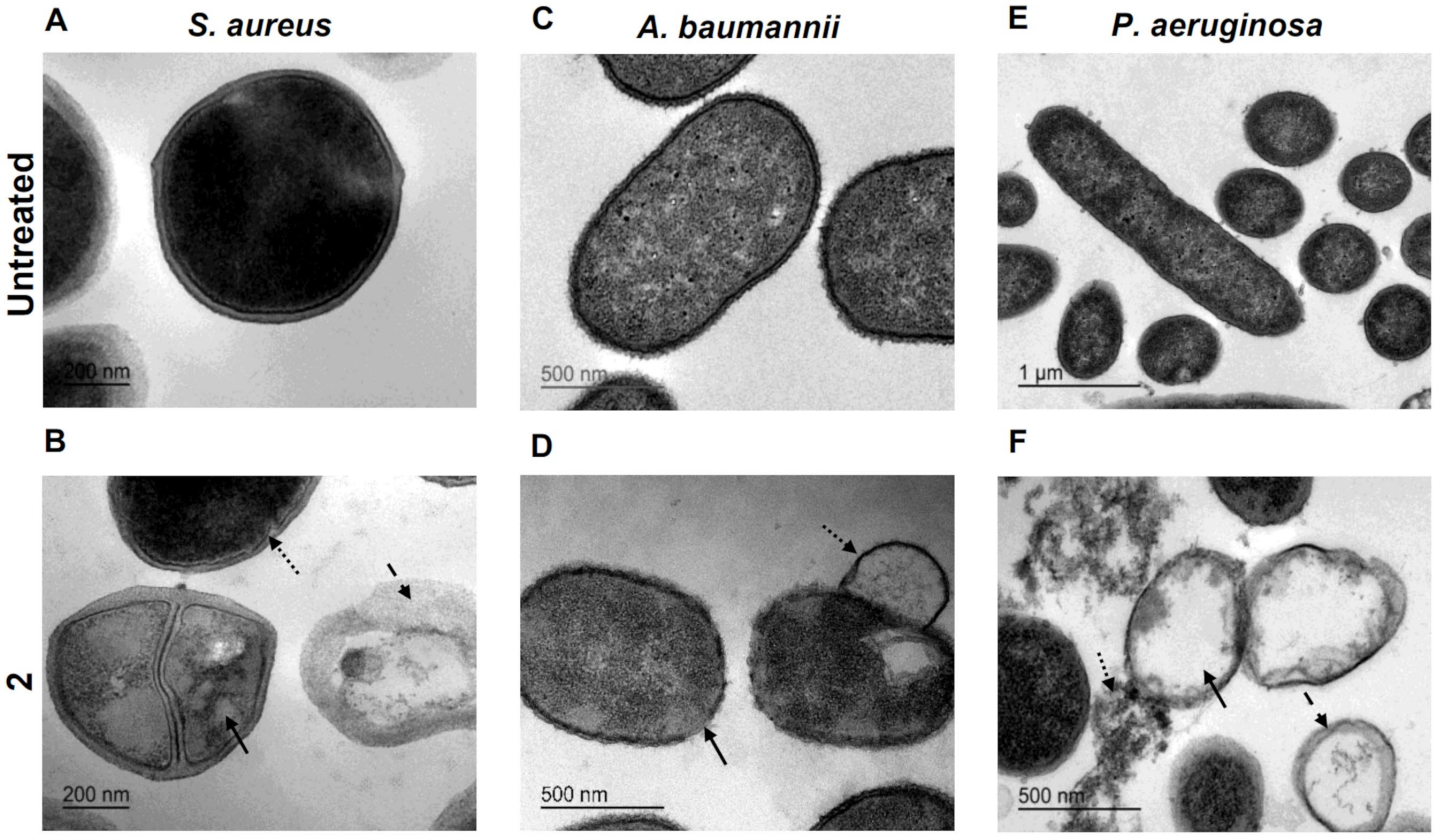
Ultrastructural analysis by TEM imaging of *S. aureus* untreated **(A)** and exposed to 2 **(B)** with arrows indicating structural damages (bar = 200 nm). *A. baumannii* untreated **(C)** and exposed to 2 **(D)** with arrows indicating structural damages (bar = 500 nm). *P. aeruginosa* untreated **(E)** (bar = 1 μm) and exposed to 2 **(F)** with arrows indicating structural damages (bar = 500 nm).

To thoroughly characterize the antimicrobial activity of complex 2, we conducted ultrastructural analysis of MRSA, *A. baumannii* and *P. aeruginosa* cells treated with MIC concentrations of the compound using transmission electron microscopy (TEM). TEM analysis revealed distinct morphological alterations in bacterial cells following treatment with complex 2 compared to untreated cells, with variations in the type of damage observed among the different bacterial species. In MRSA cells treated with complex 2 (Figure 4B), we observed abnormal cytoplasmic density (straight arrow), membrane indentations (dotted arrow) and complete release of cytoplasmic content from the disrupted cells forming ghost cells (dashed arrow). Untreated control cells (Figure 4A) displayed a typical *S. aureus* cocci shape with intact cytoplasmic membranes and homogenous intracellular density. This membrane rupture alterations were also detected in *S. aureus* for the [Au(dppta)Cl_2_] complex (Ratia et al., 2022a). *A. baumannii* cells treated with complex 2 (Figure 4D) exhibited a severe effect on the bacterial surface, with an undulating and disrupted appearance (straight arrow), also forming membrane projections (dotted arrow), unlike the intact and smooth membranes observed in control cells (Figure 4C). This is in contrast to a recent ultrastructural analysis in which auranofin monotherapy showed no impact on *A. baumannii* cells (Feng et al., 2021). In Figure 4F, we observed ghost cell structures (straight arrow) in *P. aeruginosa* cells treated with complex 2, showing complete leakage of cytoplasmic content and accumulation of ruptured cell debris (dotted arrow). The remaining membranes appeared dismembered and uneven, which was in contrast to the unaltered, non-treated cells shown in Figure 4E. This ultrastructural pattern is similar to the one described for [Au(dppta)Cl2] complex 1 over the same bacterial species (Ratia et al., 2022a). These ultrastructural changes in the bacteria may be related to the disruption of the membrane caused by the metal cations in complex 2 (Nisar et al., 2019; Paesa et al., 2023). The cationic Au(III) species present in the complex will be attracted to the negatively charged molecules in both Gram-positive and Gram-negative cell walls, leading to an imbalance of charge and surface tension across the membrane that destabilizes the cell and ultimately causes the collapse of the membrane and leakage of cytoplasmic molecules and ions (Pillai et al., 2016). Furthermore, intercalation of multiple molecules of the Au(III) complex within the bilipid layer would also cause membrane damage and cytoplasmic leakage, an effect that has been described previously for the antibacterial action of N,N′-olefin functionalized bis-imidazolium Au(I) cationic complexes (Samanta et al., 2013).

### 3.4. Quantification of complex 2 bacterial uptake

**Figure 5:**
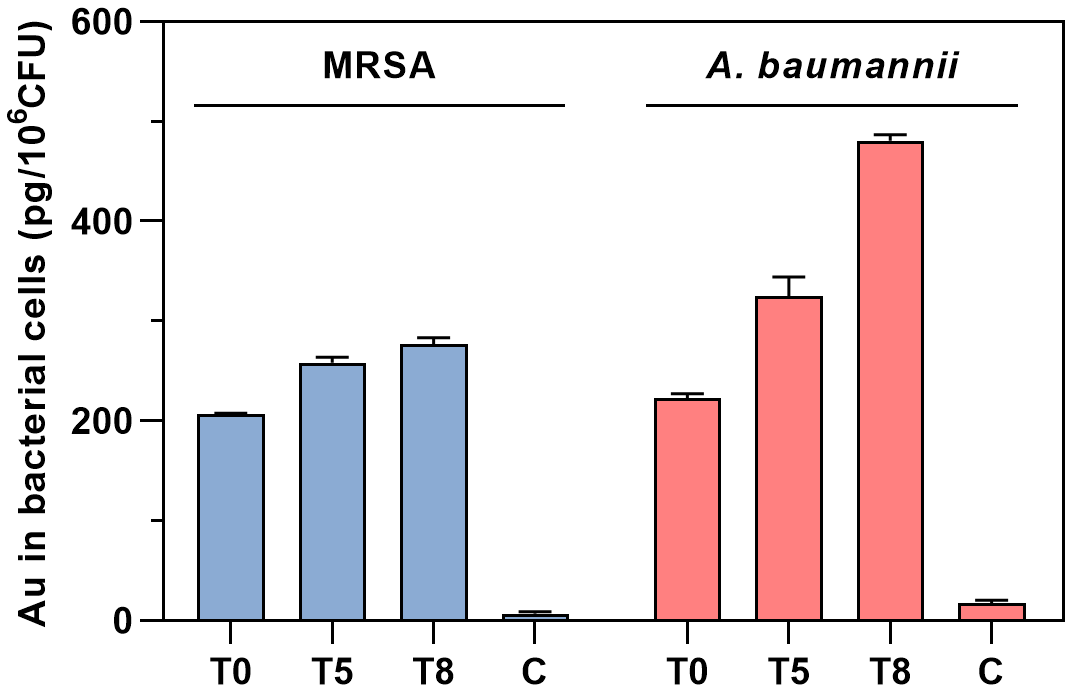
Time-dependent quantification of Au uptake in MRSA and *A. baumannii* after treatment with complex 2 analyzed by ICP-MS. T, time of sampling in minutes. C, untreated control.

The intrabacterial accumulation of complex **2** was quantified by measuring the amount of Au present in treated samples of MRSA 163501-000 and *A. baumannii* AbCr17 through ICP-MS analysis (Figure 5). The Au uptake was evaluated over time at 0, 5, and 8 min after incubation to minimize treatment-induced lysis and avoid saturation of the accumulation pathway (Prochnow et al., 2019). In both species, we detected significant amounts of Au at the beginning of the exposure, indicating an immediate internalization of complex **2**. Interestingly, the amount of Au detected in *A. baumannii* cells increased over time, doubling the initial intracellular Au content after 8 min. In contrast, the MRSA strain displayed a more restrained increase in the Au signal. Therefore, the fast internalization of complex **2** in both MRSA and *A. baumannii* cells is likely due to electrostatic interactions between the negatively charged bacterial surface and the cationic metal center (Samanta et al., 2013).

### 3.5. Antibiofilm activity

Biofilm-related infections are frequently associated with an increase in resistance to treatment and recurrence, i.e., in cystic fibrosis patients (Lebeaux et al., 2014). Novel antimicrobial agents that target biofilms are particularly valuable. In this study, we evaluated the antibiofilm activity of complex **2** by two different approaches: (i) inhibiting biofilm formation on the pre-adhesion phase (MBIC determination), and (ii) eradicating pre-formed mature biofilms (MBEC determination). Complex **2** inhibited biofilm formation at low concentrations among the *S. aureus* and *S. epidermidis* strains (MBIC 1.2–2.4 μM), only 2-fold higher than their MIC in planktonic phase (Table 3). A similar pattern was observed in gram-negative strains, with MBICs 1 to 3-folds higher than their corresponding MIC. Complex **2** had a remarkable biofilm-inhibitory effect against the *A. baumannii* and *S. maltophilia* isolates (MBIC 9.7 μM). Compared to [Au(dppta)Cl_2_] complex **1** (Ratia et al., 2022a), complex **2** exhibited significantly lower MBIC values in both gram-positive and gram-negative strains, affecting bacterial attachment to the surface more effectively. Pintus et al. (2017) also reported an important interfering effect of an Au(III) 1,2-dithiolene cyclometallated complex over *S. aureus* biofilm formation at low micromolar concentrations. Moreover, the addition of Au(III) with N-donor ligands during the pre-adhesion phase of *P. aeruginosa* reduced biofilm growth by 50% (Radulović et al., 2018), confirming the potential of Au(III) complexes to prevent biofilm formation in medical devices.

**Table 3:** Antibiofilm activity of complex 2 against a panel of biofilm-forming clinical isolates: MBIC and MBEC values, along with the corresponding percentage of biofilm eradication achieved by the treatment.

Strain ID	Species	[**2**] μM	
		MBIC	MBEC (% eradication ± SD)
163501-000	*S. aureus*	1.2	78.0 (98% ± 0.5%)
FG03015	*S. epidermidis*	2.4	156.0 (97.5% ± 0.89%)
895	*S. maltophilia*	9.7	78.0 (99% ± 0.15%)
166097-953	*P. aeruginosa*	78.0	>312.0
AbCr17	*A. baumannii*	9.7	156.0 (96.3% ± 0.45%)
HCB0093	*K. pneumoniae*	39.0	>312.0
3	*S. enterica*	19.5	312.0 (97.7% ± 0.91%)
42	*B. cepacia* complex	39.0	>312.0

The color coding denotes the potency of the concentrations - low (green), moderate (yellow) or high (red).

Regarding the eradicating activity of complex **2** over pre-formed biofilms (Table 3), we observed a > 95% decrease in bacterial metabolic activity after a 24 h treatment at 78 μM against *S. aureus* and *S. maltophilia* biofilms. For *S. epidermidis, A. baumannii*, and *S. enterica* we obtained MBEC values of 156–312 μM. However, complex **2** was ineffective against pre-formed biofilms of *P. aeruginosa*, *K. pneumoniae*, and *B. cepacia* complex. Mononuclear Au(III) complexes have been reported to disrupt 40–60% of the biofilm biomass of *P. aeruginosa* strains, although no complete eradication was observed (Savić et al., 2016). Au(I) auranofin has displayed a 1-log reduction in bacterial viability on *S. aureus* biofilms after a short period of treatment at high concentrations (Torres et al., 2016). Although the scarcity of data on the activity of Au complexes against pre-formed biofilms and the limitations of the quantification technique in detecting metabolically inactive cells make it challenging to draw definitive conclusions, our results highlight the efficacy of complex **2** at micromolar concentrations against biofilms formed by *S. aureus, S. maltophilia*, *S. epidermidis, A. baumannii*, and *S. enterica.* This suggests that complex **2** has potential as an antibiofilm agent against a range of bacterial species.

### 3.6. Synergistic antibacterial effect of complex 2 and clinical antibiotics

To enhance the efficacy of complex **2,** particularly against Gram-negative pathogens, we investigated its potential in combination with clinical antibiotics from various classes. We conducted checkerboard array experiments to assess the combination effects of complex **2** with AMK, AMP, CST, CIP, GEN, LZD, and RIF against susceptible and MDR gram-positive and gram-negative strains from the panel (Table 4). Our results indicate that complex **2** displayed synergistic effects when combined with protein synthesis inhibitors AMK and GEN (aminoglycosides) in both gram-negative and gram-positive species, with FICI values below 0.5 for most strains. This synergistic effect was also observed with LZD, another protein synthesis inhibitor, against a MRSA strain. When combined with CST, a membrane permeabilizing agent, complex **2** demonstrated a synergistic effect against *S. maltophilia*, *E. coli* and *A. baumannii* strains, with FICI values below 0.5, including the CST resistant *A. baumannii* AbCr17 and the *E. coli* extended spectrum beta-lactamase producer EC11. Regarding *P. aeruginosa*, an additive effect was observed in both resistant and susceptible strains. However, no enhancement or disturbance of inhibitory activity was observed when complex **2** was combined with CIP, a DNA gyrase inhibitor, or with RIF, a DNA-dependent RNA polymerase inhibitor. The FICI values were 2, indicating indifference. Antagonism was not detected in any of the combinations tested.

**Table 4:** Evaluation of the combination between complex 2 and clinical antibiotics against MDR and drug-susceptible strains by checkerboard assay.

Strain ID	Species	Complex 2													
		+ AMK		+ AMP		+ CST		+ CIP		+ GEN		+ LZD		+ RIF	
		FICI	Effect	FICI	Effect	FICI	Effect	FICI	Effect	FICI	Effect	FICI	Effect	FICI	Effect
163501-000	MRSA	0.37	**SYN**	1	ADD	n.a.	-	n.a.	-	0.28	**SYN**	0.5	**SYN**	2	IND
ATCC^®^ 29213	*S. aureus*	0.75	ADD	1	ADD	n.a.	-	n.a.	-	0.25	**SYN**	0.75	ADD	2	IND
166097-953	*P. aeruginosa*	0.37	**SYN**	n.a.	-	0.56	ADD	2	IND	0.25	**SYN**	n.a.	-	1	ADD
ATCC^®^ 27853	*P. aeruginosa*	0.37	**SYN**	n.a.	-	0.62	ADD	2	IND	0.25	**SYN**	n.a.	-	2	IND
895	*S. maltophilia*	0.37	**SYN**	0,62	ADD	0.14	**SYN**	2	IND	0.25	**SYN**	n.a.	-	2	IND
E11	*E. coli*	0.37	**SYN**	2	IND	0.5	**SYN**	2	IND	0.37	**SYN**	n.a.	-	2	IND
ATCC^®^ 25922	*E. coli*	0.37	**SYN**	2	IND	0.5	**SYN**	2	IND	0.75	ADD	n.a.	-	0.62	ADD
AbCr17	*A. baumannii*	0.31	**SYN**	2	IND	0.1	**SYN**	2	IND	0.5	**SYN**	n.a.	-	2	IND
ATCC^®^ 19606	*A. baumannii*	0.37	**SYN**	2	IND	0.5	**SYN**	2	IND	0.5	**SYN**	n.a.	-	1	ADD

FICI, Fractional Inhibitory Concentration Index; n.a., Not applicable; SYN, Synergy; ADD, Additive; IND, Indifference.

**Figure 6:**
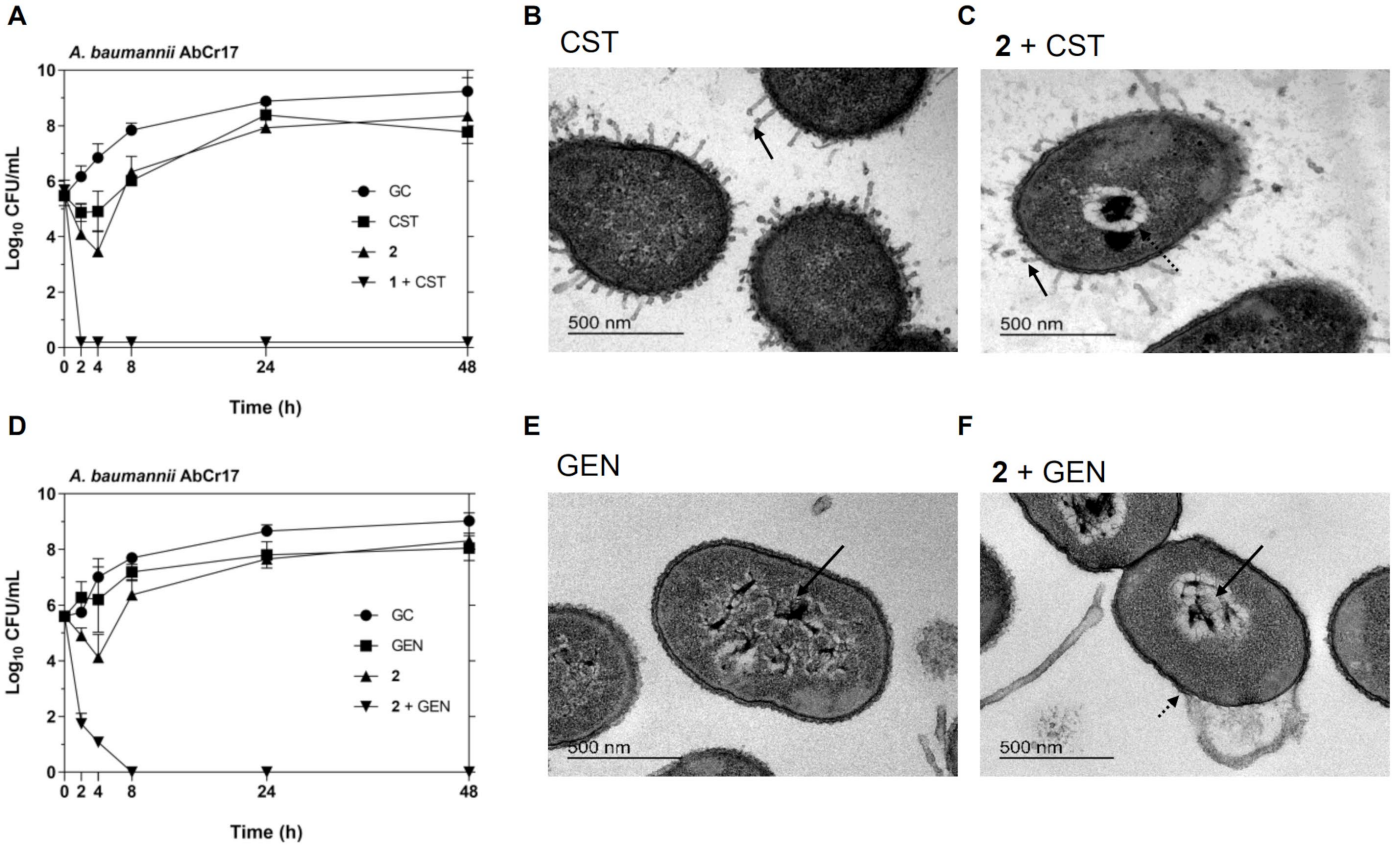
Synergistic antibacterial activity of complex 2/CST and complex 2/GEN combination against *A. baumannii* AbCr17 strain by time-kill kinetics and TEM imaging. **(A)** Time-kill kinetics of 2 in combination with CST. Graph shows the lowest bactericidal combination: CST at 0.03x MIC and 2 at 0.125x MIC. GC, Growth control. **(B)** *A. baumannii* cells treated with CST. **(C)** *A. baumannii* cells treated with complex 2/CST combination showing arrows indicating structural damages (bar = 500 nm). **(D)** Time-kill kinetics of 2 in combination with GEN. Graph shows the lowest bactericidal combination: GEN at 0.25x MIC and 2 at 0. 25x MIC. GC, Growth control. **(E)** *A. baumannii* cells treated with GEN. **(F)** *A. baumannii* cells treated with complex 2/GEN combination showing arrows indicating structural damages (bar = 500 nm).

To further characterize the synergistic effect of complex **2** with CST and aminoglycosides, we performed a time-dependent killing assay against the CST- and GEN-resistant *A. baumannii* AbCr17 strain and analyzed the ultrastructural damages of the combinations using TEM imaging (Figure 5). In the time-dependent killing assays, we observed a bactericidal synergistic effect of complex 2 and CST at concentrations below the MIC (CST = 0.86 μM; **2** = 0.6 μM) in less than 2 h. Both CST and **2** curves in monotherapy follow a similar trend as the untreated control (Figure 6A). The complex **2**/GEN combination also showed a synergistic bactericidal effect after 8 h at concentrations four times below the MIC of both drugs, with no sign of rebound growth in the following time points (Figure 6D). TEM images showed that CST-treated cells (Figure 6B) had minor protrusions across the cell wall with an almost intact cytoplasm. With the **2**/CST combination (Figure 6C), we observed protrusions (straight arrow) and cytoplasmic retraction (dotted arrow), which may be attributed to alterations in the cytoskeletal matrix. A similar effect on the membrane has been described for the combination of auranofin and CST against *A. baumannii* and *P. aeruginosa* (Feng et al., 2021). In *A. baumannii* treated with GEN we detected clear signs of DNA coagulation and nucleoid aggregation (straight arrow) (Figure 6E). This effect was enhanced using the **2**/GEN combination (straight arrow) (Figure 6F), which also showed significant membrane damage, causing indentations on the cell wall (dotted arrow).

Overall, the synergistic effect observed between complex **2** and CST or GEN greatly enhances the activity of complex **2** against Gram-negative pathogens at lower concentrations. Notably, the inhibitory concentration of complex **2** is reduced 2 to 4-fold, when combined with CST or GEN respectively, in the AbCr17 pan-resistant strain, leading to a re-sensitization of the bacteria to antibiotic treatment. This re-sensitizing effect is of particular importance when treatment options are limited, as in the case of MDR strains. A similar effect has been observed for auranofin in combination with CST in MDR strains (Sun et al., 2020).

The permeability barrier of the outer membrane has been related to the lack of activity of other Au complexes like auranofin against gram-negative strains (Thangamani et al., 2016). CST binds to the lipopolysaccharides of the Gram-negative outer membrane, causing membrane disruption and lytic bacterial death. Therefore, it has an optimal application in combination therapy to facilitate the uptake of Au-based drugs across the gram-negative cell wall (Torres et al., 2018; Sun et al., 2020; Feng et al., 2021). GEN, on the other hand, acts via irreversible binding to the 30S subunit of the bacterial ribosome, inducing protein mistranslation. Although aminoglycosides like GEN do not directly target the membrane, they can affect membrane composition through the incorporation of mistranslated membrane proteins into the cytoplasmic membrane, thereby increasing cell permeability (Kohanski et al., 2007). Alternatively, aminoglycosides can activate the redox-responsive two component systems that produce reactive oxygen species (ROS) via tricarboxilic acid (TCA) cycle (Ren et al., 2019). In our study, we hypothesize that the combination of **2** with the membrane-permeabilizing agents CST or GEN may increase the cellular uptake of the Au(III)-DTC complex, facilitating its interaction with intracellular targets and/or magnifying the disrupting effect on the bacterial membrane, ultimately accelerating bacterial cell death events.

### 3.7. Assessment of resistance development

**Figure 7:**
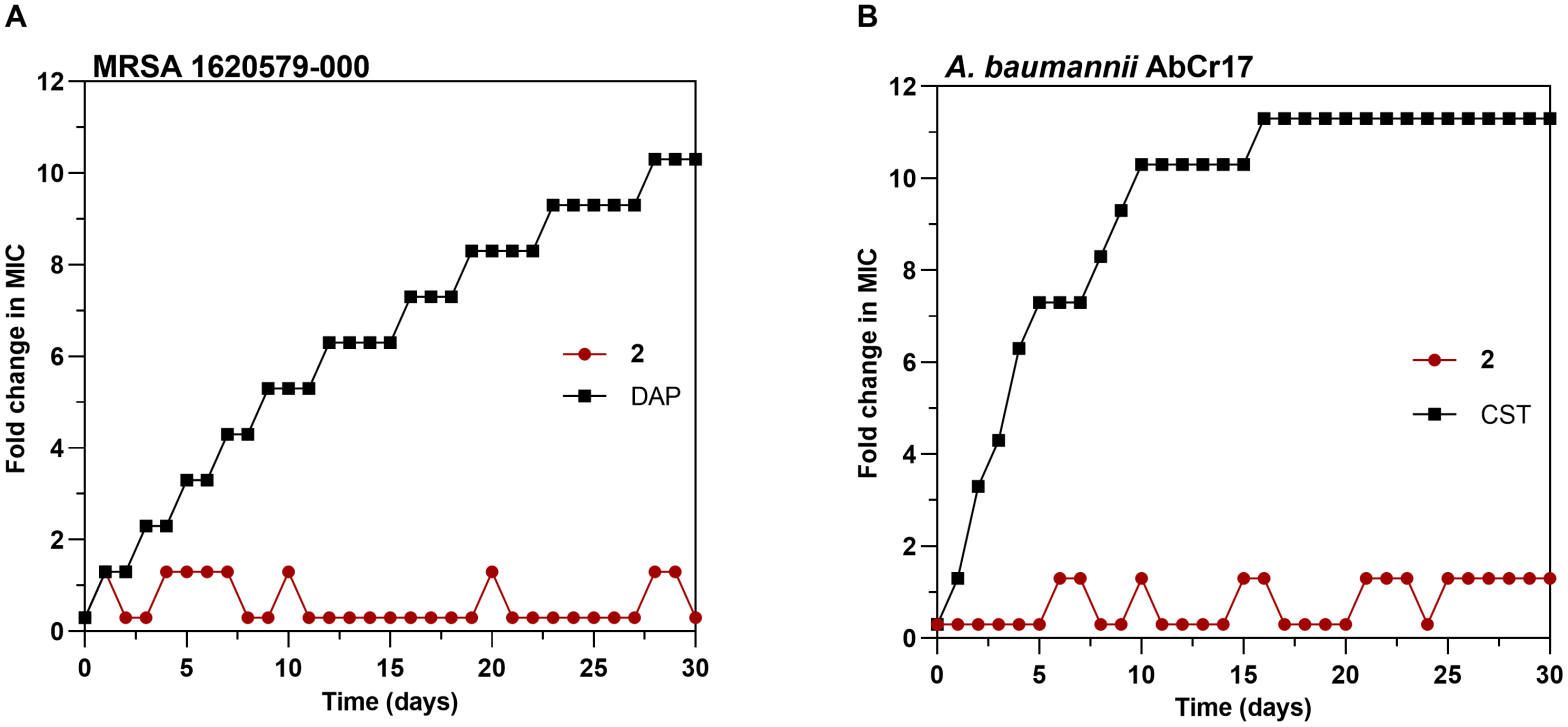
Development of resistance to complex 2 in **(A)** MRSA 1620579-000 strain and **(B)** *A. baumannii* AbCr17 strain with daptomycin (DAP) and colistin (CST) controls, respectively. The y axis shows the fold increase in the MIC value after each daily serial passage compared to the parental strain. For DAP, the highest concentration tested was 256x MIC, and for CST, the highest concentration tested was 2048x MIC. Graphs are representative of two independent experiments.

To explore the probability of resistance development to complex **2**, we favored the selection of genetic resistance to **2** by 30-day sequential step culturing. These sequential cultures were performed under sub-inhibitory concentrations of **2** to force selective pressure. No changes in the initial MIC for **2** of MRSA 163501-000 (Figure 7A) or *A. baumannii* AbCr17 (Figure 7B) were detected after 30-daily passages under sub-MIC levels. In contrast, resistant mutants for DAP (in MRSA) and CST (in *A. baumannii*) appeared at day 2 of sub-MIC culturing, reaching MICs of DAP/CST 10-fold higher than the parental strain by the end of the experiment. The lack of complex **2**-resistant mutants in a 30-day timeframe suggests that complex **2** has multiple or non-specific bacterial targets, which severely reduces the chance of developing resistance (Conlon et al., 2015). These results are consistent with the difficulties observed in selecting genetic resistance to C^N and C^S cyclometallated Au(III) complexes (Büssing et al., 2021; Ratia et al., 2022a) as well as for auranofin (Harbut et al., 2015), which may indicate that complex **2** mechanism of action is not easily bypassed by the bacteria.

### 3.8. Transcriptomic analysis by RNA sequencing

**Figure 8:**
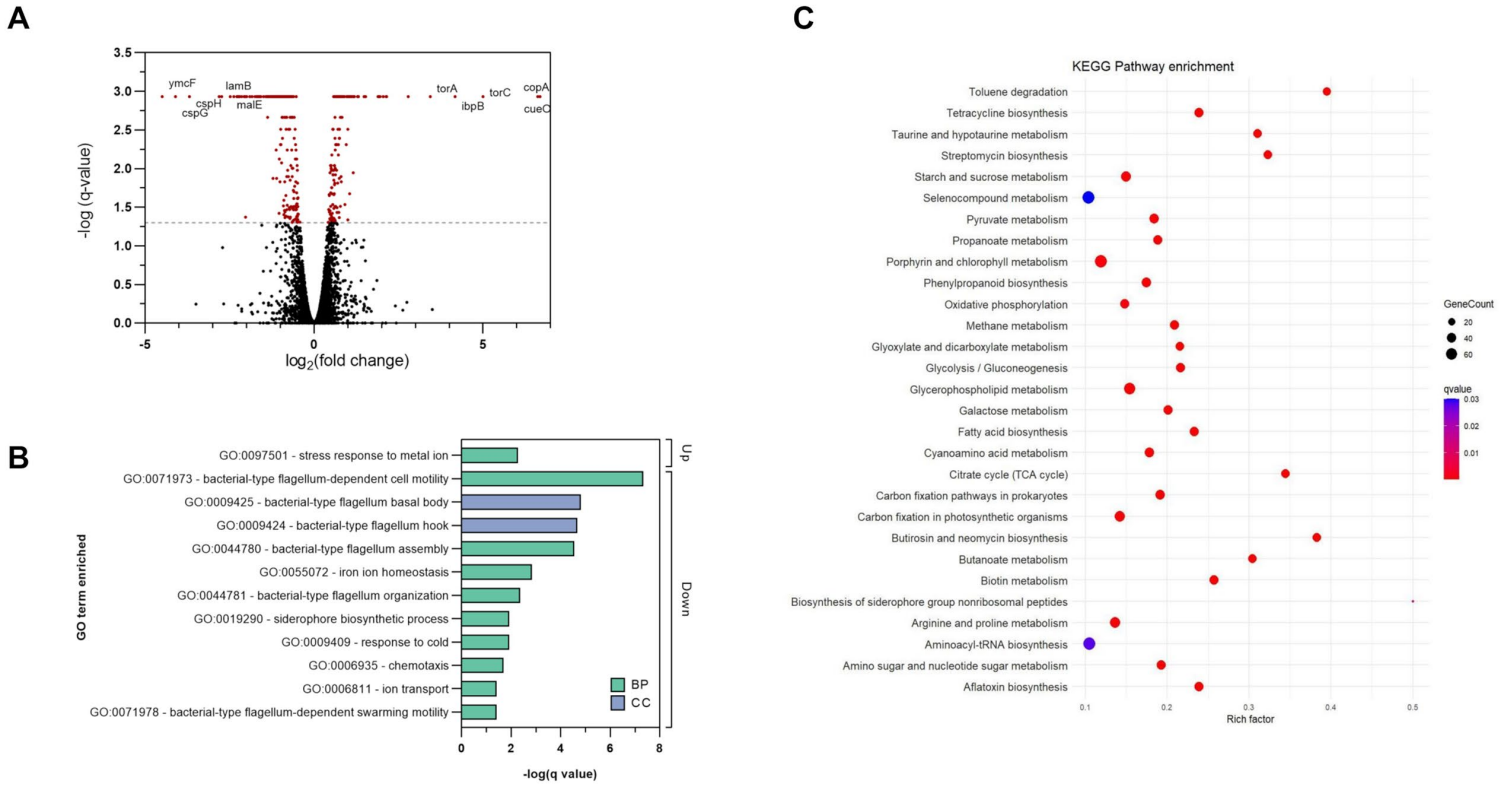
Transcriptomic analysis of complex 2 -treated *E. coli* samples compared to untreated controls. **(A)** Volcano plot showing DEGs. The dotted line represents the significance threshold set at q-value <0.05, and significant DEGs are colored in red. **(B)** GO analysis of significant DEGs induced by complex 2. Bars indicate the q-value of the enriched GO terms. Color coding indicates the type of GO classification. BP, biological process. CC, Cellular component. **(C)** Enriched KEGG pathways based on DEGs. Color coding indicates the q-value of the enriched pathways. Rich factor represents the ratio of DEGs in the pathway to the total number of genes annotated in that pathway.

To better understand the antibacterial mechanism of complex **2** and the bacterial adaptation to its action, we analyzed the differentially expressed genes (DEGs) of treated and untreated samples of *E. coli* at a transcriptome level. *E. coli* cultures were exposed to sub-inhibitory concentrations of **2** during log-phase to prevent from excessive killing and obtain high quality RNA extractions. DEG analysis showed that complex **2** treatment altered the transcriptomic levels of 388 genes, among which 155 were up-regulated and 233 were down-regulated compared to the untreated sample. The volcano plot in Figure 8A shows the DEG pattern in the complex **2**-treated sample. The top significantly up-regulated DEGs included: *copA*, which encodes for a metal-translocating ATPase involved in metal resistance; *cueO*, an oxidase involved in metal homeostasis and protection against oxidative stress; trimethylamine-*N*-oxide reductase (*torA*, *torC*) and heat-shock proteins (*ibpB*). Interestingly, the metal transporter *copA* as well as other heat-shock proteins have also appeared as one of the most upregulated DEGs in *B. subtilis* after treatment with a C^N-cyclometallated Au(III) complex (Chakraborty et al., 2021). According to Gene Ontology (GO) enrichment analysis based on DEGs, the most significantly up-regulated biological process was associated with stress response to metal ion (Figure 8B), likely caused by the bacterial reaction to the Au ion. The top significantly down-regulated DEGs included cold-shock proteins related to purine metabolism and peptidoglycan biosynthesis (*cspB*, *cspH*, *cspG*); hypothetical genes (*ymcF*), maltoporin (*lamB*) and maltose binding protein (*malE*). Both *lamB* and *malE* are key elements of the maltodextrin pathway, responsible for the internalization and degradation of maltodextrins into the gram-negative cytoplasm and are involved in trehalose biosynthesis (Dumont et al., 2019). Trehalose has been shown to contribute to *E. coli* outer membrane stability in front stress (Purvis et al., 2005) and together with the downregulation of peptidoglycan biosynthesis genes, gives further evidence of the indirect effect of complex **2** on the bacterial membrane by impairing the mRNA translation of this gene. The downregulation of *lamB* has also been associated previously with resistance to tetracycline in *E. coli* (Xu et al., 2006). Among the significantly down-regulated GO processes, many are associated with motility, membrane transport and homeostasis (Figure 8B). The KEGG pathway enrichment analysis of the significant DEGs identified a total of 29 enriched pathways comparing complex **2**-treated versus control samples, represented in Figure 8C. Notably, under complex **2** exposure we observed an alteration of the TCA cycle, which has an essential function in energy production and synthesis of biosynthetic precursors. It has been demonstrated that bactericidal antibiotics enhance the production of ROS by using internal iron from Fe-S clusters to promote hydroxyl radical formation through the Fenton reaction (Kohanski et al., 2007). With a total of five enzymes with Fe–S clusters, the TCA cycle is a pathway most sensitive toward ROS, leading to antibiotic-induced oxidative stress and antimicrobial lethality (Cheng et al., 2006). The glyoxylate and dicarboxylate metabolism pathway, an alternative to the TCA cycle that is also associated with gluconeogenesis, was enriched upon exposure to complex **2**. This pathway is critical for acetate and fatty acid metabolism in bacteria, plays a significant role in pathogenesis, and is up-regulated under oxidative or antibiotic stress conditions (Ahn et al., 2016). In addition, other energy metabolism pathways like the pyruvate, propanoate and galactose metabolism were also dysregulated in complex **2**-treated samples. We also observed an enrichment in pathways more related to membrane composition and stability induced by complex **2** like fatty acid biosynthesis and glycerophospholipid pathways. Fatty acid biosynthesis has been reported to increase after ciprofloxacin exposure (Li et al., 2018) and has been validated as an antibiotic target by drugs like isoniazid or triclosan (Wright and Reynolds, 2007). The glycerophospholipid pathway produces metabolites that provide stability, fluidity and permeability of bacterial membranes in front of stress and prevent cell injury (Xia et al., 2019). Below the top 20 enriched pathways, the starch and glucose metabolism also appeared significantly altered. This pathway is responsible for trehalose biosynthesis and is linked to the downregulation of maltoporin, as well as sharing similarities with the transcriptomic effects of an Au(I) N-heterocyclic carbene compound recently described (Wang et al., 2022). Other KEGG pathways related to specific antibiotic biosynthesis, degradation of benzene homologs or taurine metabolism were also found enriched. Overall, our transcriptomic analysis revealed changes in important energy metabolism pathways like the TCA cycle and glyoxylate metabolism, as well as in membrane-stability related pathways, which appear to be crucial in the bacterial adaptation process in complex **2** induced toxicity.

### 3.9. Inhibition of bacterial thioredoxin reductase

**Figure 9:**
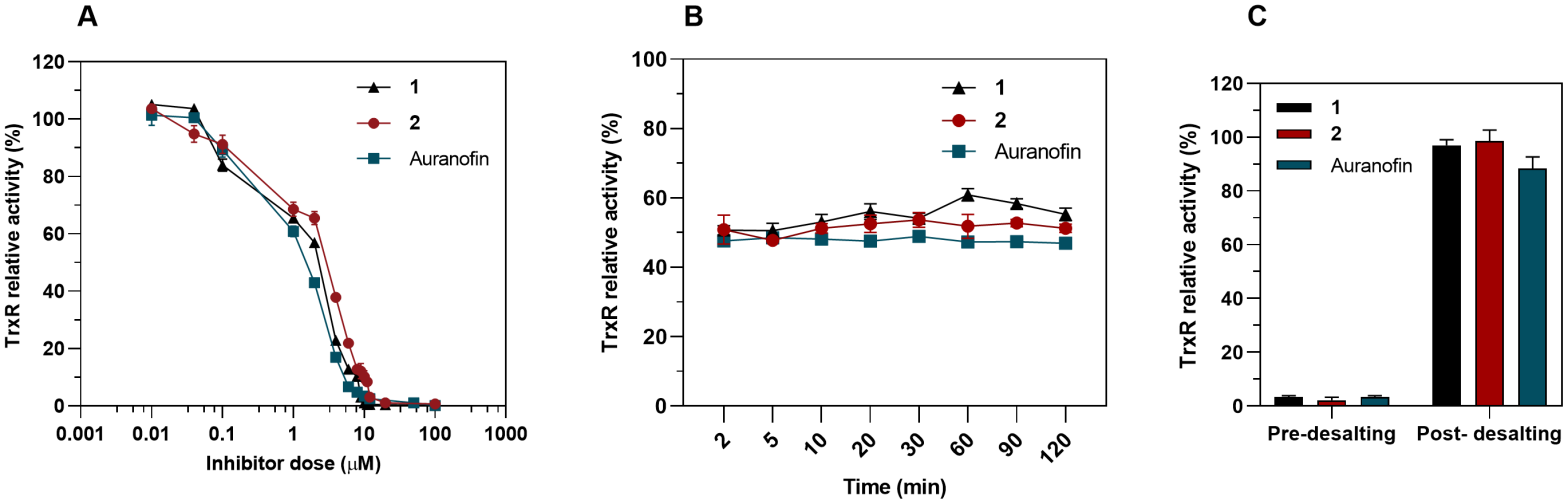
Inhibition of bacterial TrxR via DTNB assay. **(A)** Dose-dependent inhibition assay of 1, 2 and auranofin as a control. Graph shows dose–response for the TrxR relative activity after a 30 min exposure to treatment (Mean ± SD). **(B)** Time-dependent inhibition assay. TrxR was preincubated with a constant concentration of 1 (IC_50_ = 1.33 μM), 2 (IC_50_ = 1.92 μM) and auranofin (IC_50_ = 1.11 μM) and TrxR activity was measured at different time points. **(C)** 50 nM of bacterial TrxR were incubated 30 min with 10 μM of complex 1, 2 or auranofin and the enzymatic activity was measured before and after removing the Au complexes through a desalting column (Mean ± SD).

We hypothesized that Au(III) complex **2**, along with its related complex **1**, could act as an inhibitor of bacterial TrxR, considering that TrxR is the primary bacterial target for auranofin and related Au(I) complexes (Harbut et al., 2015). To assess the inhibition of pure recombinant *E. coli* TrxR, we conducted a Trx-coupled DTNB reduction assay. Our results showed that both **1** and **2** acted as strong inhibitors of TrxR in a concentration-dependent manner, with IC_50_ values of 1.33 and 1.92 μM (R^2^ = 0.96) respectively, which is similar to auranofin (IC_50_ = 1.11 μM) (Figure 9A). While auranofin is a slightly stronger inhibitor of *E. coli* TrxR compared to **2**, it is not very effective against *E. coli* based on its MIC values. This indicates that antibacterial activity does not necessarily correlate with the strength of inhibition of the pure bacterial TrxR enzyme system. The TrxR inhibitory activity of complex **1**, **2** and auranofin remained constant after incubation with the IC_50_ through the entire time-course assay, suggesting the action as competitive rather than irreversible inhibitors (Figure 9B). After removing the Au complex from the reaction through a Zeba Spin 40 K desalting column (ThermoFisher Scientific), almost 99% of the TrxR activity was restored for complexes **1** and **2** and 88% for auranofin (Figure 9C), indicating reversible inhibition (Lu et al., 2013). Recently, Büssing et al. (2021) demonstrated the potent inhibition of the *E. coli* TrxR by an Au(III) *N*-heterocyclic carbene, further validating TrxR as an intracellular target for Au(III) complexes. Overall, complex **2,** as well as the previously described complex **1**, effectively inhibits bacterial TrxR in a concentration-dependent manner, most likely through competitive and reversible inhibition. Targeting TrxR with Au(III) compounds provides an unconventional mechanism of action that is not covered by current antibiotics but has been validated by several other compounds with antimicrobial properties (Ren et al., 2019; Felix et al., 2021).

### 3.10. Molecular docking

**Figure 10:**
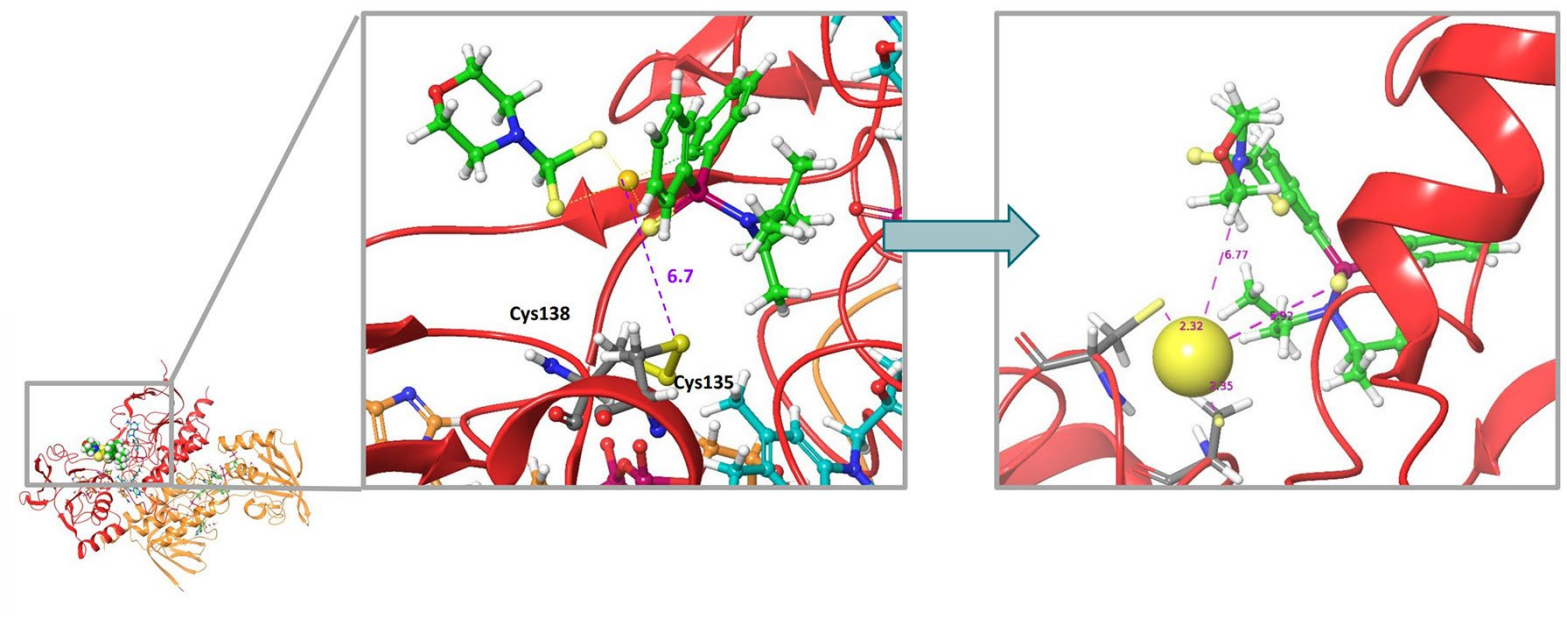
Molecular docking 3D model illustrating the most stable structure of complex 2 inside the active site of the *E. coli* TrxR. The left panel shows the rigid GLIDE docking conformation. On the left, QMMM optimization of the complex 2-bound conformation.

Rigid molecular docking coupled to Quantum Mechanics Molecular Mechanics (QM/MM) simulations were used to obtain plausible binding site conformations of complex **2** to target the bacterial homodimer TrxR. The *E. coli* TrxR pdb structure (1F6M) was prepared satisfactorily with Maestro Prepwizard, complex **2** stable conformation was initially modeled by Glide rigid docking and further refined with QM/MM simulations to match available experimental data for auranofin (Ilari et al., 2012). The most stable computed 3D structure of complex **2** inside the active site of the *E. coli* TrxR is shown in Figure 10. According to QM/MM calculations, complex **2** may be able to interact with the catalytic disulfide bridge Cys135 - Cys138, which could correlate with the inhibitory activity observed experimentally. A strong binding to the active site of the bacterial TrxR through molecular docking has only been reported for Au(III) complexes with L-histidine- containing dipeptides (Warzajtis et al., 2017). However, there must still be an equilibrium with compound, or gold, in solution, considering that we found complex **2** to be a reversible inhibitor of the enzyme, with full recovery of enzyme activity upon desalting.

### 3.11. Inhibition of human selenoprotein TrxR

**Figure 11:**
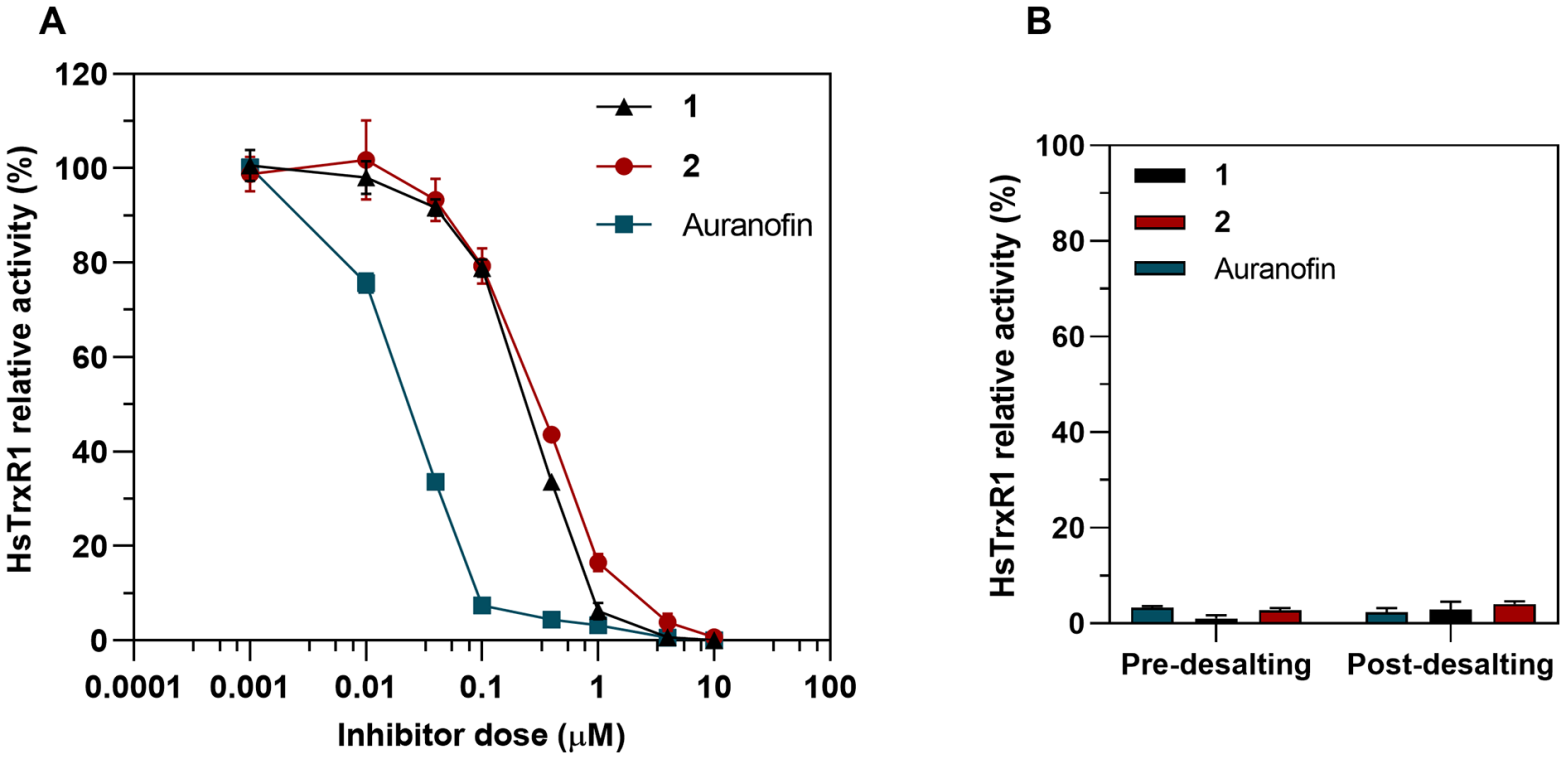
Inhibition of human TrxR1 via DTNB assay. **(A)** Dose-dependent inhibition assay of 1, 2 and auranofin as a control. Graph shows dose–response for the HsTrxR1 relative activity after a 30 min exposure to treatment (Mean ± SD). **(B)** 25 nM of HsTrx1 were incubated with 10 μM of complex 1, 2 or auranofin and the enzymatic activity was measured before and after removing the Au complexes through a desalting column.

Because the bacterial and human TrxR enzymes belong to different classes of flavoproteins, with the human enzyme furthermore being a selenoprotein containing a highly nucleophilic selenocysteine (Sec) residue that reacts directly with the gold atom of auranofin (Pickering et al., 2020; Gencheva et al., 2022), we next assessed inhibition of pure human TrxR1 (HsTrxR1). This analysis showed that complex **1**, complex **2** and auranofin were all potent inhibitors of the enzymes, albeit with auranofin being 10-fold more potent than complexes **1** and **2** (Figure 11A). In contrast to the inhibition of *E. coli* TrxR, the human enzyme was irreversibly inhibited as reflected by retained inhibition upon desalting through a Zeba Spin 40 K desalting column (ThermoFisher Scientific) (Figure 11B). This pattern of inhibition agrees with the proposed covalent targeting by the gold compounds of the catalytic Sec residue in this enzyme (Pickering et al., 2020; Gencheva et al., 2022). Because the development of new antibacterial agents requires that their cytotoxicity profiles toward mammalian cells of complexes and tissues are less potent than toward the bacterial cells, we next assessed the cytotoxicity profiles of complexes **1** and **2**.

### 3.12. *In vitro* toxicity in mammalian cells

To evaluate the potential cytotoxicity of complex **2**, we tested its effects on two liver cell lines: tumoral HepG2 and non-tumoral THLE-2, and compared it to auranofin. After a 72-h treatment, complex **2** showed an IC_50_ value of 33 μM in the non-tumoral THLE-2 cell line. Complex **2** was more potent against the tumoral Hep G2 cell line (IC_50_ = 14.2 μM), as expected due to the known antiproliferative effect of Au(III) compounds. In comparison, auranofin showed cytotoxicity in both Hep G2 (IC_50_ = 3.22 μM) and THLE-2 (IC_50_ = 1.21 μM) cell lines, indicating it is approximately 10 times more toxic than complex **2**. This is noteworthy, especially considering the improved antibacterial activity of complex **2** compared to auranofin, particularly against gram-negative strains (Table 2). Overall, these results suggest that complex **2** has limited toxicity at therapeutic concentrations in mammalian cell lines, indicating the possibility of a therapeutic safety window for *in vivo* studies.

### 3.13. *In vivo* acute toxicity

As a preliminary *in vivo* toxicity evaluation, we first compared the acute toxicity of 2 mg/Kg and 5 mg/Kg doses of complex **2** with vehicle and untreated control groups. The mice were administered a single intravenous dose and monitored for 14 days. No significant weight loss, signs of toxicity or mortality were observed in the study group during the experiment compared to the control groups. After necropsy, we detected no significant differences in the organ weight/body weight (OW/BW) ratios of the main organs. Similar lack of acute toxicity has been reported previously for anticancer Au(III) dtc complexes at comparable doses (Nardon et al., 2014). Notably, the tested concentrations in the *in vivo* assay are considerably higher than the effective antimicrobial range, indicating a potential therapeutic safety window for complex **2** *in vivo*. Our results encourage further *in vivo* characterization of complex **2** as a potential antimicrobial drug.

## 4. Conclusion

In this study, we evaluated the (C^S)-cyclometallated Au(III) dithiocarbamate complex **2** as a potential organometallic antibacterial agent. This complex demonstrated remarkable redox stability in biological conditions and in the presence of biological reductants. It also exhibited strongest antibacterial activity against gram-positive isolates, including MRSA, *S. epidermidis* and *S. pneumoniae,* and was moderately active against gram-negative pathogens, showing a bactericidal effect in both cases. This selective effect was also observed in the biofilm inhibition and eradication activities of complex **2**. Such preference for gram-positive bacteria has already been described for other Au complexes and may be linked to the strong inhibition of the bacterial TrxR observed, since gram-positive bacteria lack GSH and rely only on a functional Trx system for redox homeostasis. On the other hand, the ultrastructural membrane damage observed in TEM images and the bacterial uptake could suggest that complex **2** directly interacts with the bacterial cell wall, potentially via destabilization of membrane charges or through insertion of Au atoms in the lipid bilayer to increase permeabilization. Transcriptomic analysis revealed a dysregulation of both energy metabolism pathways related to oxidative stress and membrane stability pathways, and together with its inability to produce resistant mutants, indicates that the mechanism of antibacterial action of complex **2** is likely multimodal, including inhibition of bacterial TrxR and disruption of the bacterial membrane via electrostatic interactions or indirectly via repression of genes involved in their synthesis and stability. Furthermore, complex **2** exhibited a remarkable synergistic effect against gram-negative pathogens in combination with clinical antibiotics, offering a promising therapeutic alternative for MDR gram-negative bacteria. Lastly, complex **2** demonstrated lower cytotoxicity compared to auranofin in mammalian cell lines and no acute *in vivo* toxicity signs, suggesting that it has the safety potential for further *in vivo* studies to evaluate its potential as a therapeutic agent.

In conclusion, the results of this study provide important insights into the potential of the (C^S)-cyclometallated Au(III) dithiocarbamate complex **2** as a scaffold for developing novel metalloantibiotics with efficacy against MDR pathogenic bacteria, and warrants further investigation.

## Data availability statement

The datasets presented in this study can be found in online repositories. The names of the repository/repositories and accession number(s) can be found in the article/Supplementary material.

## Ethics statement

The Animal Experimentation Ethical Committee of the University of Barcelona (CEEA 82/16) approved all the procedures described in this study.

## Author contributions

SS, CR, and FL-O: conceptualization. CR, VB, RS, YG, MA, MI, and QC: methodology. CR: statistical analysis. SS, CR, FL-O, RS, and EA: writing—original draft preparation. CR, FL-O, RS, EA, FL, and SS: writing—review and editing. All authors have read and agreed to the published version of the manuscript.

## Funding

This work was funded by the Planes Nacionales de I + D + i 2008–2011/2013–2016 and 2020–2022 (PID2019-106658RB to FL), Instituto de Salud Carlos III (PI19/00478 PI22/00148 to SS), Subdirección General de Redes y Centros de Investigación Cooperativa, Ministerio de Economía y Competitividad, Spanish Network for Research in Infectious Diseases (REIPI RD12/0015/0013 and REIPI RD16/0016/0010 to SS) co-financed by the European Development Regional Fund “A way to achieve Europe,” operative program Intelligent Growth 2014–2020, and Plan Propio of the University of Almería (PPUENTE2020/007). MA is recipient of a fellowship from the Spanish MINECO (BES-2014-069237). We acknowledge support from the grant CEX2018-000806-S funded by MCIN/AEI/ 10.13039/501100011033, and support from the Generalitat de Catalunya through the CERCA Program. EA acknowledges funding support from Karolinska Institutet, The Knut and Alice Wallenberg Foundations (KAW 2019.0059), The Swedish Cancer Society (21 1463 Pj), The Swedish Research Council (2021-02214), The Cayman Biomedical Research Institute (CABRI), National Laboratories Excellence program under the National Tumor Biology Laboratory project (2022-2.1.1-NL-2022-00010) and the Hungarian Thematic Excellence Programme (TKP2021-EGA-44) and The National Research, Development and Innovation Office (NKFIH) grant ED_18-1-2019-0025. SS belongs to the SGR group 2021SGR01569 from the Generalitat de Catalunya (Catalonia, Spain).

## Conflict of interest

SS, CR, RS, MI, and FL-O have patents on complex **1** and **2**. QC and EA are shareholders of Selenozyme AB selling recombinant selenoproteins, including human TrxR. EA is a shareholder of Thioredoxin Systems AB, developing antibiotics targeting the bacterial thioredoxin system.

The remaining authors declare that the research was conducted in the absence of any commercial or financial relationships that could be construed as a potential conflict of interest.

## Publisher’s note

All claims expressed in this article are solely those of the authors and do not necessarily represent those of their affiliated organizations, or those of the publisher, the editors and the reviewers. Any product that may be evaluated in this article, or claim that may be made by its manufacturer, is not guaranteed or endorsed by the publisher.

## Footnotes

^1^ https://www.ncbi.nlm.nih.gov/assembly/GCF_000743255.1
