# Supplementary material for: Novel gold(III)-dithiocarbamate complex targeting bacterial thioredoxin reductase: antimicrobial activity, synergy, toxicity, and mechanistic insights
Source: Front Microbiol. 2023 Jun 2;14:1198473. doi: 10.3389/fmicb.2023.1198473 (PMC10272563; doi:10.3389/fmicb.2023.1198473)
Supplement: Supplementary file 1 [file Table_1.DOCX]

Supplementary Material

Novel gold(III)-dithiocarbamate complex targeting bacterial thioredoxin reductase: antimicrobial activity, synergy, toxicity and mechanistic insights

**Carlos Ratia^1^, Victoria Ballén^1^, Yaiza Gabasa^1^, Raquel G. Soengas^2^, María Velasco de Andrés^3^, María José Iglesias^4^, Qing Cheng^5^, Francisco Lozano^3,6,7^, Elias S.J. Arnér^5,8^, Fernando López-Ortiz^4*^, Sara M. Soto^1,9*^**

^1^Barcelona Institute for Global Health (ISGlobal), Universitat de Barcelona, Barcelona, Spain.

^2^Organic and Inorganic Chemistry Department, University of Oviedo, Oviedo, Spain.

^3^August Pi i Sunyer Biomedical Research Institute (IDIBAPS), Barcelona, Spain.

^4^Área de Química Orgánica, Centro de Investigación CIAIMBITAL, Universidad de Almería, Spain.

^5^Division of Biochemistry, Department of Medical Biochemistry and Biophysics, Karolinska Institutet, SE-171 77 Stockholm, Sweden.

^6^Servei d'Immunologia, Centre de Diagnòstic Biomèdic, Hospital Clínic de Barcelona, Spain.

^7^Departament de Biomedicina, Facultat de Medicina, Universitat de Barcelona, Barcelona, Spain

^8^Department of Selenoprotein Research and the National Tumor Biology Laboratory, Budapest, Hungary.

^9^CIBER Enfermedades Infecciosas (CIBERINFEC), Instituto de Salud Carlos III, Madrid, Spain.

**Actual affiliations:** Carlos Ratia: Thioredoxin Systems AB; Raquel G. Soengas: Organic and Inorganic Chemistry Department, University of Oviedo, Oviedo, Spain.

*** Correspondence:**Sara M. Soto
sara.soto@isglobal.org

Fernando López-Ortiz
flortiz@ual.es

# Supplementary Data

**Table S1: Protein and synthetic DNA sequence for production and purification of *E. coli* TrxR and Trx**

| **Gene** | **Protein Sequence** | **Synthetic DNA sequence** |
| --- | --- | --- |
| ***E. coli TXNRD1*** | MGSSHHHHHHGTMSDSEVNQEAKPEVKPEVKPETHINLKVSDGSSEIFFKIKKTTPLRRLMEAFAKRQGKEMDSLRFLYDGIRIQADQTPEDLDMEDNDIIEAHREQIGGMGTTKHSKLLILGSGPAGYTAAVYAARANLQPVLITGMEKGGQLTTTTEVENWPGDPNDLTGPLLMERMHEHATKFETEIIFDHINKVDLQNRPFRLNGDNGEYTCDALIIATGASARYLGLPSEEAFKGRGVSACATCDGFFYRNQKVAVIGGGNTAVEEALYLSNIASEVHLIHRRDGFRAEKILIKRLMDKVENGNIILHTNRTLEEVTGDQMGVTGVRLRDTQNSDNIESLDVAGLFVAIGHSPNTAIFEGQLELENGYIKVQSGIHGNATQTSIPGVFAAGDVMDHIYRQAITSAGTGCMAALDAERYLDGLADAK- | ATGGGCAGCAGCCATCATCATCATCATCACGGTACCATGTCGGACTCAGAAGTCAATCAAGAAGCTAAGCCAGAGGTCAAGCCAGAAGTCAAGCCTGAGACTCACATCAATTTAAAGGTGTCCGATGGATCTTCAGAGATCTTCTTCAAGATCAAAAAGACCACTCCTTTAAGAAGGCTGATGGAAGCGTTCGCTAAAAGACAGGGTAAGGAAATGGACTCCTTAAGATTCTTGTACGACGGTATTAGAATTCAAGCTGATCAGACCCCTGAAGATTTGGACATGGAGGATAACGATATTATTGAGGCTCACAGAGAACAGATTGGTGGTATGGGCACGACCAAACACAGTAAACTGCTTATCCTGGGTTCAGGCCCGGCGGGATACACCGCTGCTGTCTACGCGGCGCGCGCCAACCTGCAACCTGTGCTGATTACCGGCATGGAAAAAGGCGGCCAACTGACCACCACCACGGAAGTGGAAAACTGGCCTGGCGATCCAAACGATCTGACCGGTCCGTTATTAATGGAGCGCATGCACGAACATGCCACCAAGTTTGAAACTGAGATCATTTTTGATCATATCAACAAGGTGGATCTGCAAAACCGTCCGTTCCGTCTGAATGGCGATAACGGCGAATACACTTGCGACGCGCTGATTATTGCCACCGGAGCTTCTGCACGCTATCTCGGCCTGCCCTCTGAAGAAGCCTTTAAAGGCCGTGGGGTTTCTGCTTGTGCAACCTGCGACGGTTTCTTCTATCGCAACCAGAAAGTTGCGGTCATCGGCGGCGGCAATACCGCGGTTGAAGAGGCGCTGTATCTGTCTAACATCGCTTCGGAAGTGCATCTGATTCACCGCCGTGACGGTTTCCGCGCGGAAAAAATCCTCATTAAGCGCCTGATGGATAAAGTGGAGAACGGCAACATCATTCTGCACACCAACCGTACGCTGGAAGAAGTGACCGGCGATCAAATGGGTGTCACTGGCGTTCGTCTGCGCGATACGCAAAACAGCGATAACATCGAGTCACTCGACGTTGCCGGTCTGTTTGTTGCTATCGGTCACAGCCCGAATACTGCGATTTTCGAAGGGCAGCTGGAACTGGAAAACGGCTACATCAAAGTACAGTCGGGTATTCATGGTAATGCCACCCAGACCAGCATTCCTGGCGTCTTTGCCGCAGGCGACGTGATGGATCACATTTATCGCCAGGCCATTACTTCGGCCGGTACAGGCTGCATGGCAGCACTTGATGCGGAACGCTACCTCGATGGTTTAGCTGACGCAAAATAA |
| ***E. coli TRX*** | MGSSHHHHHHGTMSDSEVNQEAKPEVKPEVKPETHINLKVSDGSSEIFFKIKKTTPLRRLMEAFAKRQGKEMDSLRFLYDGIRIQADQTPEDLDMEDNDIIEAHREQIGGMSDKIIHLTDDSFDTDVLKADGAILVDFWAEWCGPCKMIAPILDEIADEYQGKLTVAKLNIDQNPGTAPKYGIRGIPTLLLFKNGEVAATKVGALSKGQLKEFLDANLA- | ATGGGCAGCAGCCATCATCATCATCATCACGGTACCATGTCGGACTCAGAAGTCAATCAAGAAGCTAAGCCAGAGGTCAAGCCAGAAGTCAAGCCTGAGACTCACATCAATTTAAAGGTGTCCGATGGATCTTCAGAGATCTTCTTCAAGATCAAAAAGACCACTCCTTTAAGAAGGCTGATGGAAGCGTTCGCTAAAAGACAGGGTAAGGAAATGGACTCCTTAAGATTCTTGTACGACGGTATTAGAATTCAAGCTGATCAGACCCCTGAAGATTTGGACATGGAGGATAACGATATTATTGAGGCTCACAGAGAACAGATTGGTGGTATGAGCGATAAAATTATTCACCTGACTGACGACAGTTTTGACACGGATGTACTCAAAGCGGACGGGGCGATCCTCGTCGATTTCTGGGCAGAGTGGTGCGGTCCGTGCAAAATGATCGCCCCGATTCTGGATGAAATCGCTGACGAATATCAGGGCAAACTGACCGTTGCAAAACTGAACATCGATCAAAACCCTGGCACTGCGCCGAAATATGGCATCCGTGGTATCCCGACTCTGCTGCTGTTCAAAAACGGTGAAGTGGCGGCAACCAAAGTGGGTGCACTGTCTAAAGGTCAGTTGAAAGAGTTCCTCGACGCTAACCTGGCGTAA |
| His-tag: underlined letter  SUMO sequence: green letter  Stop codon (TAA): highlighted in turquoise | | |
